# Mutant KRAS Reprograms Luminal Progenitor Differentiation to Establish a Novel Transformation-Competent Mammary Epithelial Cell State

**DOI:** 10.3390/cancers18213483

**Published:** 2026-10-28

**Authors:** Madison N. Wicker, Hye Kyung Lee, Kerry Vistisen, Kay-Uwe Wagner

**Affiliations:** 1 Department of Oncology and Center for Molecular Medicine and Genetics, Wayne State University School of Medicine, Barbara Ann Karmanos Cancer Institute, Detroit, MI 48201, USA; wickerm@karmanos.org (M.N.W.);; 2 National Institute of Diabetes, Digestive and Kidney Diseases, National Institutes of Health, Bethesda, MD 20892, USA; hyekyung.lee@nih.gov

**Keywords:** mammary gland, cell differentiation, triple negative breast neoplasms, mammary tumor virus, FLP recombinase, proto-oncogene proteins p21(ras), green fluorescence protein, GFP, flow cytometry, single-cell sequencing, scRNA-seq, particle-templated instant partition sequencing, PIP-seq

## Abstract

Background/Objectives: Previous studies reported that basal-like breast cancers may originate from luminal progenitors, suggesting that a classification of this malignancy is not strictly indicative of its cellular origin. Reinforcing this notion, we recently provided experimental evidence in genetically engineered models that basal-like and claudin-low mammary tumors, the two most common triple-negative breast cancer (TNBC) types, can evolve from transformed luminal epithelial cells. The primary objective of this study was to define, at the single-cell level, the earliest molecular changes associated with neoplastic transformation prior to the formation of mammary cancer. Methods: We isolated green fluorescent protein (GFP)-labeled mammary epithelial cells from *MMTV-Flp FSF-Kras^G12D^ Rosa26^FSF-GFP^* mice and controls without mutant KRAS by fluorescence-activated cell sorting (FACS) and performed particle-templated instant partition sequencing (PIP-seq). Following hierarchical clustering of the single-cell transcriptomes, as well as differential gene expression and gene ontology analyses, we used Slingshot, TSCAN, and Monocle3 to infer developmental trajectories of luminal epithelial cell states. Results: Based on their single-cell transcriptomes, GFP-expressing mammary epithelial cells were predominantly luminal-type and resolved into 11 transcriptionally distinct cellular states organized into six functional groups. Most notably, mutant KRAS induced a novel oncogenic-permissive state with unique molecular features, including cancer-associated metabolic reprogramming, oncogenic pathway activation, and epithelial-to-mesenchymal cell plasticity. Trajectory inference positioned this KRAS-associated state along an alveolar progenitor differentiation path. Conclusions: The collective results provide new insights into the epithelial states associated with early neoplastic transformation and identify signaling and metabolic programs that may present candidate targets for cancer prevention.

## Simple Summary

Gaining a better understanding of the normal epithelial cells in the mammary gland from which tumors form is critical for developing strategies to prevent breast cancer. The aim of this study was to isolate and sequence individual mammary epithelial cells from a genetically engineered mouse model that develops an aggressive breast cancer type, allowing early cellular and molecular changes to be identified before tumors form. Through this approach, we found that luminal cells exist in several distinct states and functional groups. Under the influence of the mutant KRAS oncogene, a novel epithelial state emerged with altered metabolism, activation of cancer-related signaling, and increased cellular plasticity. Bioinformatic analyses revealed that this KRAS-induced state is associated with a developmental path involving alveolar progenitors. The observed cellular and molecular changes associated with malignant transformation may contain potential candidate target genes and pathways for future cancer-prevention strategies.

## 1. Introduction

With over 2.3 million new cases annually and a projected increase to over 3 million by 2040, breast cancer will remain, worldwide, the most commonly diagnosed malignancy in women [1]. Based on histopathological and immunohistochemical (IHC) classifications, malignant breast tumors are typically invasive ductal carcinoma (IDC, 75%) or lobular carcinoma (ILC, 15%), both of which express estrogen and progesterone receptors (70–80% IDC and >90% ILC, respectively). Among hormone receptor (HR)-positive IDC cases, about 9–11% co-express Epidermal Growth Factor Receptor 2 (HER2, ERBB2/neu) [2,3]. Only 3–5% of HR-negative breast cancers overexpress ERBB2, but upregulation or amplification of this receptor is uncommon in ILCs [4]. Eleven to 15% of breast cancers, which lack both hormone receptors and ERBB2, are referred to as triple-negative (TNBC). The IHC-based characterization of tumors is generally used as a surrogate for the classification of intrinsic molecular subtypes based on genome-wide expression profiling. The latter includes HR-positive luminal A and luminal B, HER2/ERBB2-enriched, basal-like, and normal-like [5,6]. While many basal-like breast cancers are triple-negative, the TNBC subtype encompasses an extended fraction of mammary tumors that can be grouped into six subclusters, including breast cancers with mesenchymal features called claudin-low (CLBC) [7,8,9,10].

The gene expression profiles and malignant properties of HR-positive and ERBB2-overexpressing breast cancers are governed by the subtype-defining growth factor receptors. In contrast, the initiation and progression of TNBCs are typically associated with mutations in Transformation-related Protein 53 (TP53, TRP53, p53) along with alterations in molecular programs that define the basal epithelial lineage and mesenchymal features (e.g., p63, PRRX1), metabolism and cell proliferation (PI3K, c-MYC), as well as homologous recombination-mediated DNA repair (e.g., BRCA1) [9,11,12,13,14,15]. Claudin-low mammary cancers also show a robust activation of the RAS/MAPK pathway, which is particularly high in tumors with stem cell properties [16]. Notably, mutations in *KRAS* or *HRAS* and their downstream effectors are present in CLBC cell lines commonly used as experimental models to study TNBC (e.g., MDA-MB-231, Hs578T, SUM159PT) [17]. Specific features that define claudin-low TNBCs are the upregulated expression of mesenchymal markers (e.g., N-CADHERIN, VIMENTIN), the reduction in tight junction and cell adhesion proteins (i.e., CLAUDIN 3, 4, 7, and E-CADHERIN), and the decrease in luminal cell surface markers (CD24, EpCAM) [18].

CLBCs encompass mesenchymal and mesenchymal stem-like TNBCs [10], and it was therefore logical to suggest that these tumors may arise from undifferentiated mammary stem cells [19,20]. In contrast to this notion, the computational analysis of genomic datasets showed that CLBCs share features with established intrinsic molecular subtypes [16,21], suggesting that some of these malignancies may originate from luminal or ERBB2-enriched breast cancers that subsequently underwent epithelial-to-mesenchymal transition (EMT). Using advanced genetically engineered mouse models, two recent studies by our team provided experimental evidence that basal-like and claudin-low mammary tumors (i.e., the two most common TNBC subtypes) can evolve from the transformation of luminal epithelial cells within mammary ducts and alveolar units in response to oncogenic KRAS signaling [22,23]. The acquisition of basal epithelial and mesenchymal features was preceded by a hybrid state in early lesions, in which epithelial cells co-expressed luminal, basal, and mesenchymal markers. These insights into cellular plasticity during tumor initiation and progression were made possible by experimental models in which the transforming oncogene is continuously expressed in epithelial cells independent of their differentiation state [24]. Moreover, these models provided corroborating data that the maintenance of mesenchymal features in triple-negative tumor cells was dependent on the persistent expression of the cancer-initiating oncogene [22], suggesting that cellular plasticity can be reversed by inhibiting a key oncogenic driver.

In this study, we used single-cell RNA sequencing (scRNA-seq) on genetically labeled mammary epithelial cells from *MMTV-Flp FSF-Kras^G12D^ Rosa26^FSF-GFP^* experimental females and *MMTV-Flp Rosa26^FSF-GFP^* controls to investigate the earliest cellular and molecular changes in lineage plasticity prior to the initiation of triple-negative mammary cancer. Hierarchical clustering of single-cell transcriptomes revealed that the *MMTV-Flp* transgene targets GFP and mutant KRAS expression to eleven cellular states across six functional groups, mostly with luminal epithelial features. More importantly, oncogenic KRAS induces a distinct transformation-competent state characterized by oncogenic pathway activation, metabolic reprogramming, and lineage plasticity. Collectively, these results provide new insights into oncogene-induced epithelial cell plasticity and changes in signaling modalities that may serve as candidate targets for cancer prevention.

## 2. Material and Methods

### 2.1. Genetically Modified Mouse Strains

The *MMTV-Flp* [Tg(MMTV-flpo)29542Kuw] transgenic mouse line expressing the codon-optimized Flp recombinase (FLPo) under the long MMTV promoter (MMTV-SV40-BSSK backbone) was generated by our team [23]. The mouse strains that carry the targeted alleles of the *FSF-Kras^G12D^* [B6(Cg)-Kras^tm5Tyj^/J] [25] and the *Rosa26^CAG-FSF-GFP^* [Gt(ROSA)26Sor^tm1.2(CAG-EGFP)Fsh^] [26] were kind donations from the laboratories of Drs. Jacks and Fishell to The Jackson Laboratory (Stock No: 023590 and 32038-JAX, respectively). All transgenes were either generated on or backcrossed into the FVB/N genetic background for more than ten generations. The genotyping of the mouse strains was carried out by PCR using the primers listed in Supplementary Table S1. All mice were housed in pathogen-free micro-isolator cages under a 12/12-h light/dark cycle.

### 2.2. Conventional Flow Cytometry

Mammary epithelial cells (MECs) were isolated from enzymatically dissociated mammary gland tissues of MMTV-Flp *Rosa26*-GFP and MMTV-Flp FSF-*Kras^G12D^ Rosa26*-GFP mice as described below, cultured for 48 h, trypsinized, and resuspended in 1× phosphate-buffered saline (1× PBS). Cells were counted using an automated CellDrop cell counter (DeNovix Inc., Wilmington, DE, USA) and stained with EpCAM-PE, CD49f-PE/Cy7, and CD61-APC (Supplementary Table S2) for 25 min at 4 °C in the dark. Next, cells were washed with ice-cold 1× FACS buffer (1% bovine serum albumin, 0.1% sodium azide in 1× PBS) to remove unbound antibodies and pelleted by centrifugation at 900 rpm. Cells were resuspended in 1× FACS buffer and analyzed using a Cytek Northern Lights flow cytometer. Initial gates were established using forward-scatter (FSC) and side-scatter (SSC) to exclude cellular debris. Cell aggregates were excluded, and viable cells were identified using 7-aminoactinomycin (7-AAD) viability dye (Cat. #51-68981E, BD Pharmingen, San Diego, CA, USA). Within the viable mammary epithelial population, GFP-positive cells were identified, and subpopulations were characterized according to EpCAM and CD49f expression. CD61 expression was additionally evaluated within the GFP-positive MEC population. The SpectroFlo software v3.0.3 (Cytek Biosciences, Fremont, CA, USA) was used for quality control, data acquisition, and spectral unmixing on the flow cytometer. The data was exported to FlowJo v10.8.1 (Waters Corporation, Milford, MA, USA) to process, visualize, gate, compare, and statistically analyze the Flow Cytometry Standard (FCS) files.

### 2.3. Preparation of Single-Cell Suspensions of Viable GFP-Positive Mammary Epithelial Cells for PIP-Seq

The #3 and #4 mammary glands were removed during necropsy and imaged under a fluorescent stereoscope (Zeiss Discovery V8, Zeiss, Oberkochen, Germany) to confirm GFP expression in the ductal epithelium. Next, the tissues were minced into small fragments and transferred to a 50 mL conical tube containing pre-warmed digest media consisting of 20 mL DMEM/F12 buffered with 4-(2-hydroxyethyl)-1-piperazineethanesulfonic acid (HEPES), 200 µL 100 units/mL pen-strep, 300 µL 100 units/mL hyaluronidase, 1000 µL 2 mg/mL collagenase, 40 µL 100 µg/mL gentamicin, and 60 µL of 0.75 units/mL fungizone. The minced tissue was incubated at 37 °C for approximately 30–40 min, or until all visible clumps were dispersed. The dissociated mammary cells were centrifuged at 500× *g* for 5 min; the supernatant was aspirated, and the cell pellet was washed with 10 mL Hanks’ balanced salt solution (HBSS) supplemented with 5% fetal bovine serum (FBS). Cells were centrifuged at 500× *g* for 5 min, and the supernatant was discarded. The cell pellets were resuspended in 5 mL of pre-warmed TrypLE Express (Cat. #12604013, Gibco, Thermo Fisher Scientific, Waltham, MA, USA) and incubated for 3 min at room temperature. Following incubation, 10 mL of HBSS with 5% FBS was added, and cells were centrifuged at 500× *g* for 5 min. The supernatant was aspirated, and pellets were resuspended in Dispase solution (Cat. #42613-33-2, STEMCELL Technologies, Vancouver, BC, Canada) and incubated for 2 min at room temperature with gentle agitation before adding 10 mL HBSS supplemented with 5% FBS. The cell suspensions were passed through 100 µm cell strainers (Cat. #352360, VWR International–Avantor, Radnor, PA, USA) to obtain single-cell suspensions. Cells were then centrifuged at 500× *g* for 5 min and resuspended in a master mix containing 0.5 mL of 1× MACS buffer containing 4 µL of the following biotin-conjugated antibodies against CD31, CD45, CD34, and Ter119 for lineage depletion (Supplementary Table S3). Cells were incubated for 30 min at 4 °C, washed with 10 mL of 1× MACS buffer, and centrifuged at 500 rpm for 5 min. The supernatant was aspirated, and cells were resuspended in 500 µL containing 100 µL of anti-biotin microbeads (Cat. #130-090-485, Miltenyi Biotec, Bergisch Gladbach, Germany) and 400 µL of 1× MACS buffer. Cells were incubated for 20 min at 4 °C. During this incubation, MACS separation columns (Cat. #130-042-201, Miltenyi Biotec) were placed in a magnetic stand and equilibrated with 3 mL 1× MACS buffer. After the 20-min incubation, the cells were centrifuged at 2000 rpm for 5 min, and the supernatant was discarded. The sample was then resuspended in 1 mL of 1× MACS buffer and passed through a magnetized MS Column with a 30 µm separation filter. FACS tubes collected the flow-through, with unbound cells representing the lineage-negative (Lin^−^) population containing MECs. The Lin^−^ population was centrifuged at 500× *g* for 5 min; the supernatant was discarded, and the pellet was resuspended in 1 mL of Advanced DMEM/F12 with Glutamax, HEPES, and Pen/Strep. The cell count and viability were confirmed using an automated CellDrop cell counter. Cells were kept on ice before fluorescence-activated cell sorting using the Sony SH800 cell sorter (Sony Biotechnology Inc., San Jose, CA, USA). Viable cells were identified using a 7-AAD viability dye (Cat. #51-68981E, BD Pharmingen). Viable, GFP-positive cells were isolated from the Lin^−^ population and collected in a fresh FACS tube filled with 0.5 mL of 1× MACS buffer. Sorted GFP-positive mammary epithelial cells were maintained on ice and immediately processed for PIP-seq.

### 2.4. Particle-Templated Instant Partition (PIP) Sequencing

Following fluorescence-activated cell sorting, viable GFP-positive mammary cells were immediately processed for single-cell RNA-sequencing using the PIPseq T20 3′ Single Cell RNA-seq v4.0 kit (Fluent BioSciences, Watertown, MA, USA) according to the manufacturer’s protocol (Doc ID: FB0002130). Four samples were processed independently for sequencing, consisting of two replicates of pooled epithelial cells from two *MMTV-Flp FSF-Kras^G12D^ Rosa26^FSF-GFP^* experimental mice per sample and two replicates of pooled cells from six and ten *MMTV-Flp Rosa26^FSF-GFP^* control mice per sample. Cells were resuspended in partitioning reagent and loaded individually into the Particle-templated Instant Partition sequencing (PIP-seq) tubes. The PIP tubes and cells were vortexed according to the manufacturer’s protocol, thereby encapsulating individual cells within beads. Following partitioning, cells were lysed, and captured mRNA underwent reverse transcription to generate barcoded cDNA. The barcoded cDNA was subsequently amplified, isolated from the PIPs, and purified. Following amplification, the cDNA concentration was quantified using a Qubit 2.0 fluorometer (Invitrogen, Life Technologies, Carlsbad, CA, USA), and the quality and size distribution of the cDNA was analyzed using a Bioanalyzer. Samples that met quality-control criteria were then used for library preparation. The cDNA was fragmented, followed by end repair, A-tailing, and ligation of PIPseq library adapters. The library indices and corresponding adapter sequences are provided in Supplementary Table S4. The prepared cDNA libraries were amplified and purified using a magnetic bead-based cleanup protocol according to the manufacturer’s guidelines. The four library samples passed quality control for single-cell RNA-sequencing and were stored at −20 °C until sequencing. Pooled libraries were sequenced using the NovaSeqX platform (Illumina, San Diego, CA, USA) with a 25B flow cell and dual-indexed paired-end sequencing at the NIH Intramural Sequencing Center (NISC).

### 2.5. Single-Cell RNA Sequencing Analysis

Pipspeak (https://github.com/noamteyssier/pipspeak?tab=readme-ov-file accessed on 26 June 2025) was used for initial processing of the raw sequencing data. Sequence reads were subsequently aligned to the GRCm39 mouse reference genome and quantified using PIPseeker software (Fluent BioSciences, version 3.3.0). The sensitivity_3 tier filtered expression matrix was used for downstream analysis. Quality control, normalization, dimensionality reduction, clustering, UMAP visualization, and cell-state annotation were performed in R (version 4.3.3) using Seurat (version 5.3) [27]. Low-quality cells were excluded using the following thresholds: nCount_RNA > 500, nFeature_RNA > 2000 and <8000, and mitochondrial transcript percentage < 5%. Gene-expression normalization and variance stabilization were performed using SCTransform, with mitochondrial transcript percentage, total RNA counts, and number of detected genes regressed as covariates. Principal component analysis (PCA) was performed, and a shared-nearest-neighbor graph was constructed using PCs 1–30, followed by graph-based clustering at a resolution of 0.8. Uniform Manifold Approximation and Projection (UMAP) was calculated using PCs 1–30 to visualize transcriptional relationships among cells. Potential sample-associated batch effects were evaluated by visualizing the transcriptional distributions of cells across the four independently processed libraries. As an additional assessment, datasets were split by sample identity and integrated using Seurat’s CCA-based integration workflow. Initial unsupervised clustering identified 15 transcriptionally distinct cell clusters (clusters 0–14).

Cluster-specific marker genes were identified using Seurat’s FindAllMarkers function with the Wilcoxon rank-sum test, retaining only positively enriched markers with a minimum log2 fold-change of 0.5. Multiple-testing-adjusted *p* values were calculated using Bonferroni correction. The expression of selected marker genes across clusters was visualized using dot and violin plots. Original clusters 10–14 were excluded based on their non-epithelial marker profiles, and cells in original clusters 0–9 were retained for de novo epithelial re-clustering. In particular, expression of *Ptprc*, was used to identify and remove hematopoietic cells. Cluster-associated marker genes were identified by comparing cells in each epithelial cluster with cells in all remaining epithelial clusters using Seurat’s FindAllMarkers function with the default Wilcoxon rank-sum test, only.pos = TRUE, and logfc.threshold = 0.5. The returned marker tables used the default unadjusted *p*-value threshold of 0.01 and included Bonferroni-adjusted *p*-values. Library/sample identity was not included as a covariate in these tests. These analyses were used to characterize cluster-associated expression patterns rather than to establish sample-adjusted genotype effects within clusters. Following exclusion of non-epithelial populations, Seurat clusters 0–9 were subsetted and reprocessed using SCTransform, PCA, nearest-neighbor graph construction, graph-based clustering, and UMAP as described above. This re-clustering identified 11 transcriptionally distinct mammary epithelial states. Cluster-specific marker analysis was repeated using the same criteria, and the top 10 marker genes per state were visualized as a heatmap. Control and KRAS^G12D^-expressing cells were analyzed jointly to define the epithelial-state structure and were also examined separately to assess differences in the representation of epithelial states.

Module scores were calculated in Seurat using AddModuleScore with predefined gene sets for the luminal-basal identity, EMT features, and hybrid cell states. The following genes were used to distinguish luminal-basal identity: *Acta2*, *Esr1*, *Krt5*, *Krt8*, *Krt14*, *Krt18*, *Myh11*, *Pgr*, and *Prlr*. The following epithelial and mesenchymal genes were used to calculate EMT signatures: *Cdh1*, *Cdh2*, *Cldn7*, *Epcam*, *Krt8*, *Krt18*, *Ocln*, *Slug*, *Snail1*, *Twist1*, *Vim*, *Zeb1*, and *Zeb2*. Co-expression of luminal (*Krt8*) and basal (*Krt14*) markers was used to identify cells that are entering or have already assumed a hybrid state. Pathway and process enrichment analysis was performed using Metascape. Gene Ontology (GO) category significance was evaluated using the cumulative hypergeometric distribution, and q-values were calculated using the Benjamini–Hochberg procedure to account for multiple testing. Significantly enriched GO terms were further organized into seven cancer-related functional categories based on shared biological functions using Cytoscape/EnrichmentMap [28]. Additional cross-cluster GO comparisons used archived Enrichr results generated with the GO Biological Process 2021 gene-set library. For each cluster, the first 50 genes in the returned marker table were submitted without a custom background. These comparisons were descriptive and did not constitute a statistical test of differential enrichment between clusters.

To evaluate the relationships among the 11 mammary epithelial cell populations, trajectory inference and pseudotime analyses were performed using TSCAN [29], Slingshot [30], and Monocle3 [31]. For TSCAN, the Seurat object was converted to a SingleCellExperiment using the SCT assay, and Seurat cluster assignments and precomputed PCA/UMAP embeddings were retained. Cluster centroids were calculated and connected by a minimum spanning tree (MST) using TSCAN::createClusterMST; cells were mapped to MST edges and ordered in pseudotime using mapCellsToEdges and orderCells. No starting cluster was specified in TSCAN, so the package default starting node was used. TSCAN analyses were performed on all epithelial cells and separately on control and experimental cells, both with and without geometric outlier filtering. For outlier filtering, the Euclidean distance of each cell from its cluster centroid in UMAP space was calculated, and cells with a distance greater than the cluster-specific median + 3 x median absolute deviation (MAD) were excluded. Thus, outlier removal was based on within-cluster UMAP geometry rather than a separate expression-based QC threshold. Slingshot was applied to the same epithelial SingleCellExperiment objects using the Seurat cluster labels and PCA coordinates, with curve extension enabled (extend = “y”); no biological start or terminal cluster was prespecified, and lineage structure was inferred from the cluster configuration. Slingshot analyses were likewise repeated with and without the UMAP-based outlier filter. For Monocle3, the filtered Seurat object was converted to a cell_data_set and the precomputed Seurat UMAP embedding and cluster assignments were retained rather than reclustering de novo. A trajectory graph was learned with learn_graph, and cells were ordered in pseudotime using cluster #7 as the root population; no terminal endpoint was prespecified, so terminal states corresponded to leaves of the learned trajectory graph. An additional Monocle3 analysis was performed with loop closure enabled as a sensitivity analysis. Genes associated with progression along the Monocle3 trajectory were identified using graph_test on the principal graph, and results were ranked by q-value; the top 10 genes were subsequently visualized. Because the methods used different procedures for selecting or specifying the pseudotime origin, their inferred directions and pseudotime values were not treated as directly equivalent. Comparisons across methods focused on transcriptional relationships among epithelial states rather than a shared direction of differentiation. None of these analyses establishes lineage ancestry or temporal causality.

### 2.6. Mouse Mammary Tumor and Human Breast Cancer Analysis

The expression of cluster 6 signature genes was assessed using RNA-seq data sets from mutant KRAS-induced basal-like and claudin-low mammary cancers (Gene Expression Omnibus, GEO, accession number GSE157333) [22]. The Broad Institute’s Integrative Genomics Viewer (IGV) was employed to visualize the mRNA expression of signature genes and their exons. The differential expression patterns of selected genes were analyzed from breast cancer specimens in The Cancer Genome Atlas (TCGA) using the Breast Cancer Gene-Expression Miner v5.3. [32].

### 2.7. Statistical Analyses

Graphical illustrations and statistics were performed with GraphPad Prism 6 software (GraphPad Software, Inc., La Jolla, CA, USA). The tumor-free survival distributions between individually recorded animals were compared using the Log-rank test. *p* values < 0.05 (*), <0.01 (**), <0.001 (***), or <0.0001 (****) were considered statistically significant.

## 3. Results

### 3.1. Regulatory Elements of the Mouse Mammary Tumor Virus (MMTV) Target the Expression of the Flp Recombinase and Mutant KRAS to a Subset of Luminal Epithelial Progenitors

We generated a novel transgenic mouse line that expresses the Flp recombinase under the control of the Mouse Mammary Tumor Virus (*MMTV*) promoter [23]. Flp mediates the deletion of DNA fragments in the genome that are surrounded by Flp Recombination Target (*FRT*) sites, such as transcriptional *Stop* sequences (*FRT-Stop-FRT*, short *FSF*) in front of a reporter gene or an exon of an endogenous gene carrying a cancer-promoting mutation (e.g., *Kras^G12D^*; Supplementary Figure S1A). Upon Flp-mediated excision of the *Stop* sequence in the mammary epithelium of *MMTV-Flp* transgenic females, a reporter transgene or an endogenous mutant allele becomes constitutively active, independent of the functional state of particular cells as well as their normal and neoplastic descendants. By crossing the *MMTV-Flp* founder line with the *Rosa26^CAG-FSF-GFP^* reporter strain (short *Rosa26-GFP*), we previously determined that the expression of Flp recombinase was primarily targeted to cytokeratin 8 (CK8)-positive luminal epithelial cells in the mammary gland, and the green fluorescent protein (GFP) reporter was not detected in cytokeratin 14 (CK14)-expressing basal epithelial cells, fibroblasts, adipocytes, or lymphoid cells [23]. Despite the superior specificity of the *MMTV-Flp* transgene in luminal epithelial cells, the expression of GFP was highly mosaic and present within a subset of cells in the ductal epithelium [23].

**Figure 1:**
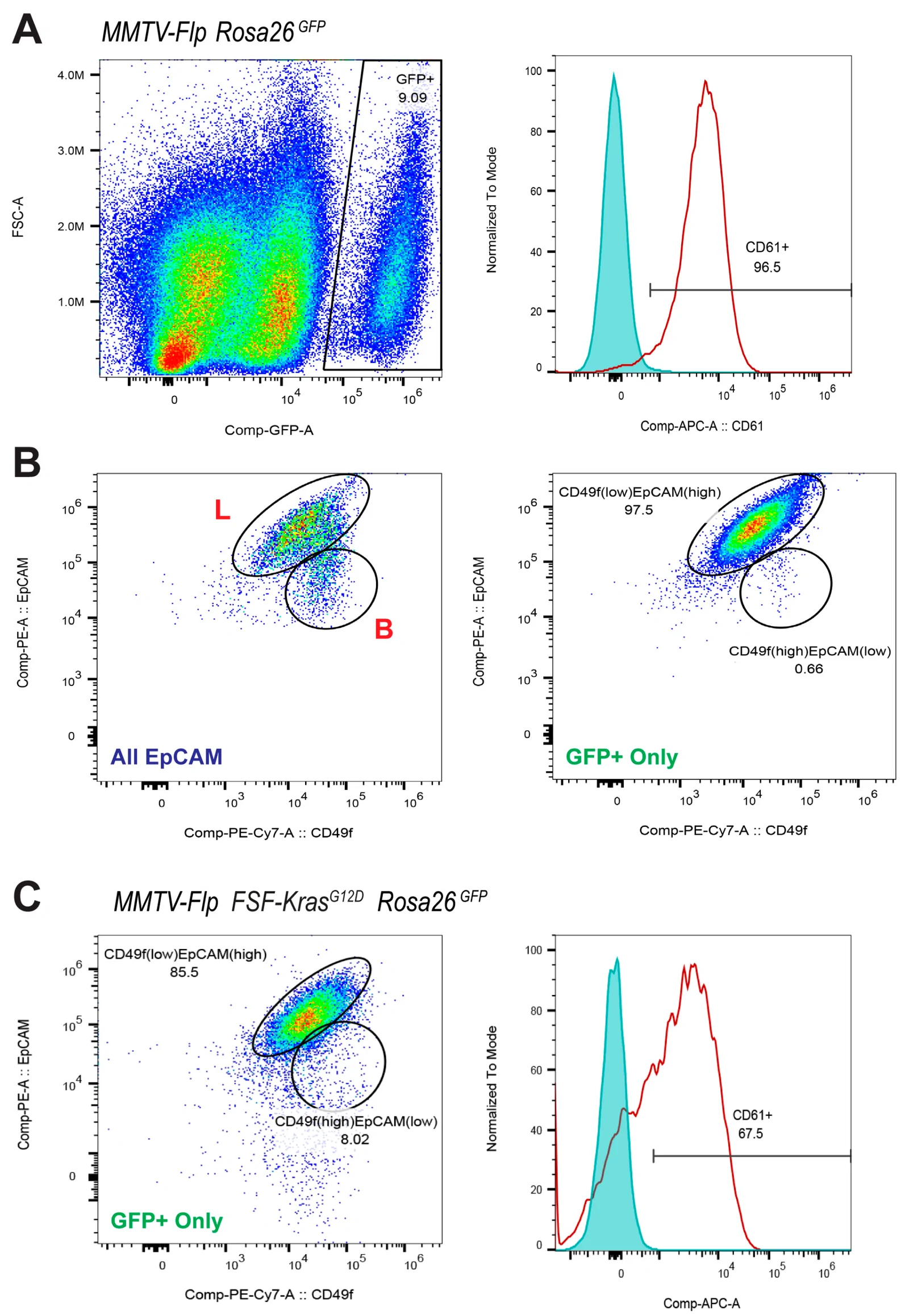
MMTV-Flp-mediated activation of GFP and mutant KRAS expression in the luminal epithelium. (**A**) Flow cytometric analysis of mammary epithelial cells from nulliparous MMTV-Flp *Rosa26*-GFP to quantify the number of GFP-expressing cells (**left**) and expression of CD61 in the subset of GFP-positive cells (**right**). (**B**) Relative proportion of luminal (L; EpCAM^hi^ CD49f^low^) and basal (B; EpCAM^low^ CD49f^high^) mammary epithelial cells expressing GFP (**right**) in comparison to all isolated epithelial cells (**left**) from MMTV-Flp *Rosa26*-GFP females. (**C**) Flow cytometric analysis of GFP-positive luminal and basal mammary epithelial cells (**left**) and CD61 expression (**right**) from nulliparous *MMTV-Flp Rosa26*-GFP *FSF-Kras^G12D^* females.

In preparation for the scRNA-seq experiment to map the developmental trajectory of normal and oncogenic-permissive mammary epithelial cells, we used flow cytometry to quantify the relative number of GFP-expressing cells from mammary gland tissues of *MMTV-Flp Rosa26-GFP* transgenic females with and without expression of oncogenic *Kras*. Following depletion of immune, erythroid, and endothelial cells using CD45-, Ter119-, and CD31-conjugated magnetic beads, the separated lineage-negative (Lin^−^) cells were labeled with antibodies against EpCAM and CD49f to mark luminal and basal mammary epithelial cells, and with CD61 to identify luminal progenitors [33,34]. The flow cytometric analysis showed that only a small fraction of viable Lin^−^ cells (approximately 9%) in *MMTV-Flp Rosa26-GFP* females were GFP-positive, but most of them (96%) clustered to the CD61-positive luminal epithelial progenitor subtype (Figure 1A). In accordance with this observation, GFP-expressing cells were confined to the luminal epithelium (97% EpCAM^high^ CD49f^low^), and less than 1% were basal epithelial cells (EpCAM^low^ CD49f^high^) (Figure 1B). We repeated the flow cytometry experiment on isolated and sorted Lin^−^ cells from mammary glands of *MMTV-Flp FSF-Kras^G12D^ Rosa26-GFP* triple transgenic females and found that these mice showed a small increase in the number of GFP-expressing basal-like epithelial cells (8% EpCAM^low^ CD49f^high^) along with a reduction in the relative number of luminal progenitors (67% CD61-positive cells) (Figure 1C). The collective results of this inaugural experiment validated that the *MMTV-Flp* transgene is expressed in a small number of luminal epithelial cells, and more specifically, luminal progenitors. Moreover, the Flp-mediated activation of mutant KRAS expression led to a minor but detectable shift from luminal progenitors towards cells with basal-like properties prior to mammary cancer formation.

### 3.2. The MMTV-Flp-Mediated Activation of Mutant KRAS in Luminal Epithelial Cell Clusters Induces a Distinct Cellular State

To characterize a small fraction of genetically labeled cells expressing the *MMTV-Flp* construct and trace their developmental trajectory in response to the activation of mutant KRAS signaling, we performed particle-templated instant partition sequencing (PIP-seq) on isolated GFP-positive mammary epithelial cells of *MMTV-Flp FSF-Kras^G12D^ Rosa26^FSF-GFP^* experimental females and *MMTV-Flp Rosa26^FSF-GFP^* controls. PIP-seq is based on high-purity single-cell partitions using hydrogel particles, permitting efficient capture, barcoding, and sequencing of rare cell populations with high sensitivity [35]. All mammary gland tissues were inspected under a fluorescence stereoscope prior to the isolation and sorting of epithelial cells to validate the expression of GFP and to ensure that the glands in KRASG12D-expressing mice did not contain overt mammary tumors (Supplementary Figure S1B). While the initiation of mammary cancer in *MMTV-Flp FSF-Kras^G12D^* females was not strictly dependent on pregnancy, the median tumor-free survival was significantly shorter in parous mice (159 days, *n* = 32) compared to nulliparous females (204 days, *n* = 41; *p* < 0.0001) (Supplementary Figure S2). Given the better predictability of tumor formation within a narrower time frame and increased likelihood of premalignant cells in the glands, we included only parous, non-pregnant females in both cohorts for this study, aged 149 to 190 days. To construct gene expression libraries from up to 20,000 cells per genotype, we sorted approximately 40,000 viable GFP-positive cells by flow cytometry from enzymatically dissociated mammary glands of multiple *MMTV-Flp Rosa26-GFP* controls and *MMTV-Flp FSF-Kras^G12D^ Rosa26-GFP* experimental females. In total, we generated and sequenced two expression libraries for each genotype, representing sorted cells from four experimental and sixteen control females according to the protocol of the PIPseqTM T20 scRNA kit v4.0 (Fluent BioSciences).

The initial examination of the gene expression data and unsupervised clustering showed that the pool of GFP-sorted cells contained 15 discrete cell types (Supplementary Figure S3A). There was a higher representation of cells from experimental females compared to controls in 14 of the 15 clusters (Supplementary Figure S3B). The initial clustering also identified a distinct cell population (cluster 7) composed predominantly of cells from mutant KRAS-expressing mice, with only one control cell assigned to this cluster (Supplementary Figure S3C). Next, we calculated module scores to discriminate epithelial subtypes and assess the expression of GFP within each of the initially identified clusters. Uniform Manifold Approximation and Projection (UMAP) analysis showed that 9 of the 15 cell populations (clusters 0–7 and 9) had high expression of luminal markers, defining a central luminal population or super-cluster (Supplementary Figure S4A, right). The basal gene expression signature was low across samples and only elevated in the small cluster 8 (Supplementary Figure S4A, left). This finding confirmed the flow cytometry results that *MMTV-Flp* expression is largely restricted to luminal epithelial cells. Expression of *Gfp* was detected in clusters 0–9, and the reporter was very low or absent in the minor subpopulations 10–14 outside the super-cluster (Supplementary Figure S4B). The more detailed analysis of individual cell lineage-specific genes revealed that cells within the central luminal and adjacent cell populations expressed genes that determined their epithelial identity, such as luminal cytokeratins (*Krt8*, *Krt18*, *Krt19*) and epithelial cell-specific adhesion molecules (*Cdh1*, *Epcam*, *Cldn3*, *Cldn4*, and *Cldn7*) (Supplementary Figure S5). Cells within clusters 10 to 14 exhibited an elevated expression of mesenchymal markers and signature genes for fibroblasts, endothelial cells, and immune cells. Most cells in these small clusters were T- and B-cells (*Cd3e*, *Cd19*), along with a few endothelial cells (*Pecam1*) (Supplementary Figure S6A) that may have exhibited strong autofluorescence during single-cell sorting, as they lacked expression of the *Gfp* reporter transcript. In subsequent analyses, we used *Ptprc* expression as an exclusion variable for most non-epithelial cells (Supplementary Figure S6B).

**Figure 2:**
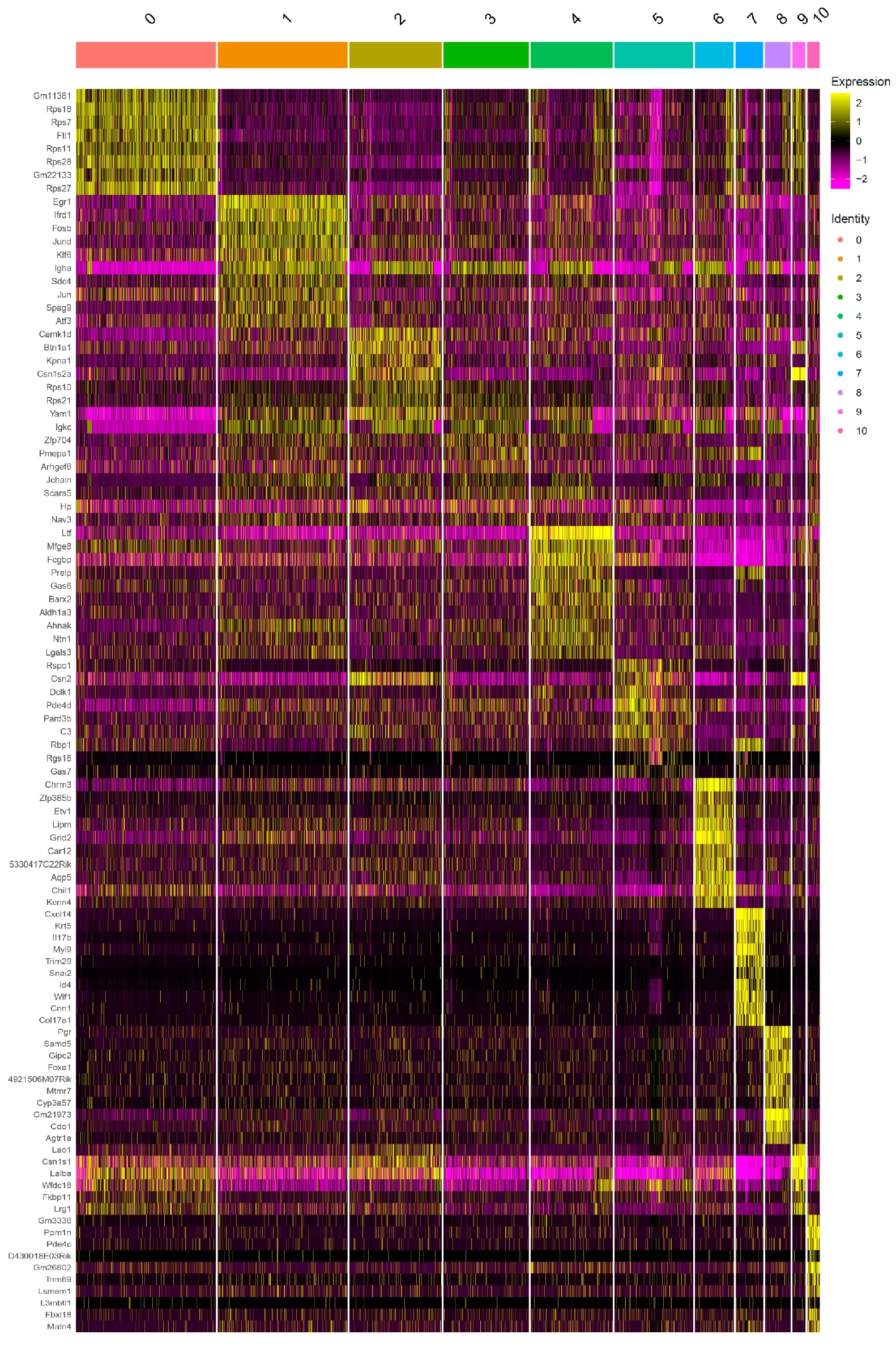
Single-cell RNA sequencing identifies eleven transcriptionally distinct epithelial cell states (clusters 0–10) targeted by the MMTV-Flp transgene. Cluster analysis of the single-cell RNA expression profiles of GFP-positive mammary epithelial cells from *MMTV-Flp Rosa26*-GFP females with and without the Flp-mediated expression of oncogenic KRAS (*FSF-Kras^G12D^*). Heatmaps show the top 10 cluster-specific marker genes defining each of the eleven mammary epithelial states.

**Figure 3:**
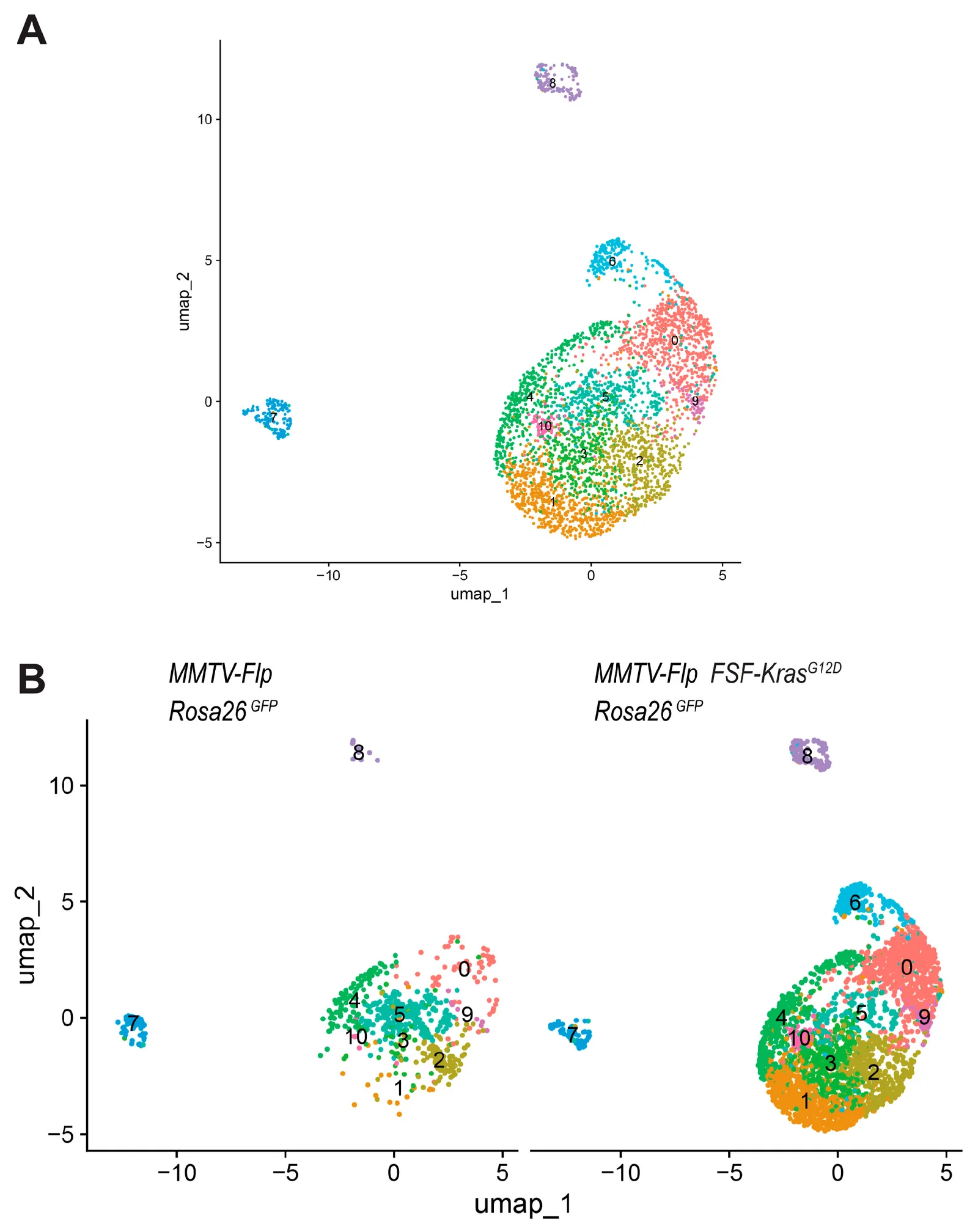
Emergence of a new cellular state (cluster 6) in close proximity to the luminal super-cluster in response to the *MMTV-Flp*-mediated activation of oncogenic KRAS. (**A**) Uniform Manifold Approximation and Projection (UMAP) plot of the eleven transcriptionally distinct mammary epithelial cell states (clusters 0–10) expressing GFP among the combined datasets from *MMTV-Flp Rosa26*-*GFP* females with and without the *FSF*-*Kras^G12D^* allele. (**B**) UMAP projection of GFP-positive epithelial cell states in control (**left**) and experimental females (**right**), revealing a unique cellular state (cluster 6) as a consequence of KRAS^G12D^ expression.

Under more stringent exclusion criteria for immune and endothelial cells, the re-clustering of the scRNA-seq data identified 11 transcriptionally distinct epithelial cell states (clusters 0–10) (Figure 2). The KRAS-enriched population initially identified as cluster 7 largely overlapped with final cluster 6 after epithelial re-clustering. Of the 207 cells in final cluster 6, 191 originated from original cluster 7 (Supplementary Figure S3). Although original cluster 7 contained one control cell, this cell was reassigned to final cluster 3; no control cells were assigned to final cluster 6. *Gfp* reporter transcripts were detected in all epithelial states (Supplementary Figure S7A), and according to UMAP visualization, clusters 0 through 6 as well as 9 and 10 formed a large common luminal super-cluster (Figure 3A). Only groups 7 and 8 appeared as separate cellular states from the super-cluster with distinctly different gene expression profiles. The annotation of the clusters as cellular states seems appropriate, since cells across all 11 epithelial populations exhibited luminal expression markers, including cluster 7, which also showed elevated expression of genes that define basal epithelial cells (Supplementary Figure S7B,C). The relative representation of epithelial states varied between control and KRAS^G12D^-expressing samples (Figure 3B). Most notably, cluster 6 was absent from the control dataset but was detected specifically in the KRAS^G12D^-expressing dataset, where it was localized adjacent to the luminal super-cluster. Cluster 6 was detected in both independently processed pooled experimental libraries, whereas no cells were assigned to this cluster in either control library (Supplementary Figures S8 and S9).

### 3.3. Transcriptional Programs Define Six Functional Groups Within Eleven Mammary Epithelial Cell States Targeted by the MMTV Promoter-Driven Flp Recombinase

**Figure 4:**
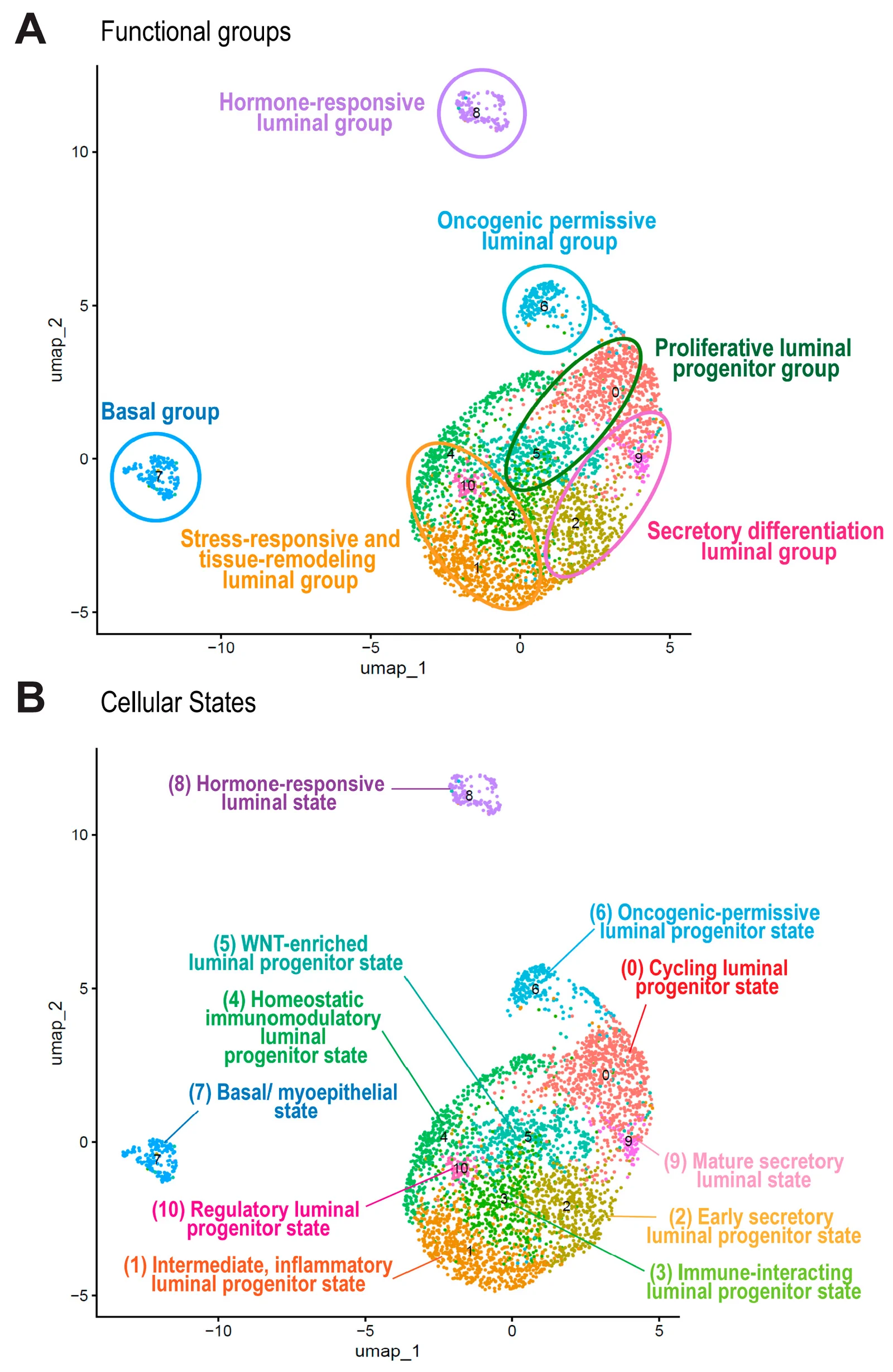
The mammary epithelial cell states targeted by the MMTV-Flp can be organized into six functional groups. (**A**) Uniform Manifold Approximation and Projection (UMAP) plot of GFP-expressing epithelial cell clusters within functional groups (encircled) as defined by the top 20 most deregulated genes within the combined single-cell RNA sequencing datasets from *MMTV-Flp Rosa26*-*GFP* females with and without the *FSF*-*Kras^G12D^* allele. (**B**) UMAP representation of GFP-positive epithelial cell clusters highlighting the cellular states defining their specific functions.

Next, we performed differential gene expression and gene ontology (GO) analyses to define distinctive features of each identified cell cluster and to organize them into functional groups. Based on cluster-specific marker expression and associated GO enrichment profiles (Supplementary Figure S10), the eleven *MMTV-Flp* target cell clusters were assembled into six functional groups (Figure 4A). The central portion of the super-cluster comprised proliferative luminal progenitors. Within this functional group, clusters 0 and 5 denote rapidly cycling luminal progenitors that express canonical luminal and proliferative transcription factors, including *Klf5*, *Sox9*, and *Gata3*. Despite these shared features, cluster 0 exhibited a higher expression of genes associated with functional differentiation (e.g., *Lalba*, *Csn3*, *Spp1*, *Areg*) and genes encoding ribosomal proteins (e.g., *Rps7*, *Rps18*, *Rps28*, *Rps11*) (Figure 4B). In contrast, cluster 5 showed enrichment for WNT signaling, expression of *Akt1*, *Stat3*, and the replication licensing factor *Mcm5*. Clusters 1, 3, 4, and 10 formed a second functional group comprising luminal cells with highly active stress-response and tissue-remodeling programs. The relative number of cells in this functional group varied between two independently prepared libraries from mutant KRAS-expressing mice (Supplementary Figure S11). This variation may reflect biological differences between the pooled samples (e.g., inter-animal variation in the oncogenic stress response), technical differences in tissue processing and cell recovery, or both. Because cells from multiple mice were pooled within each library, variation among individual animals could not be assessed. Enrichment for Activator Protein-1 (AP-1), endoplasmic reticulum (ER) stress, and differentiation programs are defining characteristics of cluster 1 as an intermediate, inflammatory luminal population. The high expression of genes associated with immunoglobulin A (IgA)-mediated immunity and apoptotic priming indicated that cluster 3 represented a luminal state engaging in crosstalk with the immune microenvironment. The specific upregulation of genes like *Egfr* and *Hey2* suggested that cells within cluster 10 may possess additional roles in response to epidermal growth factor and NOTCH signaling. As part of the functional stress-response and remodeling group, luminal epithelial cells within cluster 4 exhibited a characteristic expression of genes associated with tissue homeostasis and inflammation resolution as well as antimicrobial and immunomodulatory functions (e.g., *Mfge8*, *Ltf*).

The third functional group within the central super-cluster encompassed two cellular states with mostly secretory functions (clusters 2 and 9). Cells within cluster 2 showed moderate expression of *Kpna1*, *Csm1s2a*, *Lalba*, and *Csn2*, and high expression of ribosomal and translation-related genes, indicating that cells within this population were undergoing functional differentiation. Higher expression of *Spp1* (*Osteopontin*) and milk protein genes, along with reduced proliferative activity, signified that luminal cells in cluster 9 entered the state of secretory differentiation and turnover. Unlike the two other functional groups within the central super-cluster, cells with partial or more advanced functional differentiation in clusters 2 and 9 showed high expression of *E74-like factor 5* (*Elf5*), a master transcription factor that commits mammary epithelial cells to the alveolar lineage and milk production [36].

Among luminal epithelial cell lineages targeted by the *MMTV-Flp* transgene, cluster 8 represented a relatively small group of hormone-responsive epithelial cells outside the central super-cluster. These cells exhibited a high expression of *Epcam*, *Gata3*, *Prlr*, *Esr1*, and *Pgr*, and the presence of steroid hormone receptors and the transcriptional regulation of estrogen signaling were validated by the GO analysis. Other characteristic programs within this functional group were ion and metabolite transport. The fifth functional group and the second small population outside the central core (cluster 7) comprised cells with key features of the basal epithelial lineage, expressing *Krt5*, *Krt14*, *Acta2*, *Egfr*, and *Vim* (Supplementary Figure S7B). Cells within this cluster showed restricted enrichment for *Id4* and *Myc*, indicative of the presence of stem or progenitor cells. However, these basal cells retained expression of luminal markers (Supplementary Figure S7C), and many exhibited a dual luminal–basal expression profile score (Supplementary Figure S12). The fact that this population was not selectively enriched for females expressing mutant KRAS may indicate that basal-like and claudin-low mammary tumors do not originate from this small cell cluster, which has the most basal epithelial characteristics in the normal gland.

### 3.4. KRAS Activation Induces a Novel Oncogenic-Permissive Luminal Mammary Epithelial Cell State

**Figure 5:**
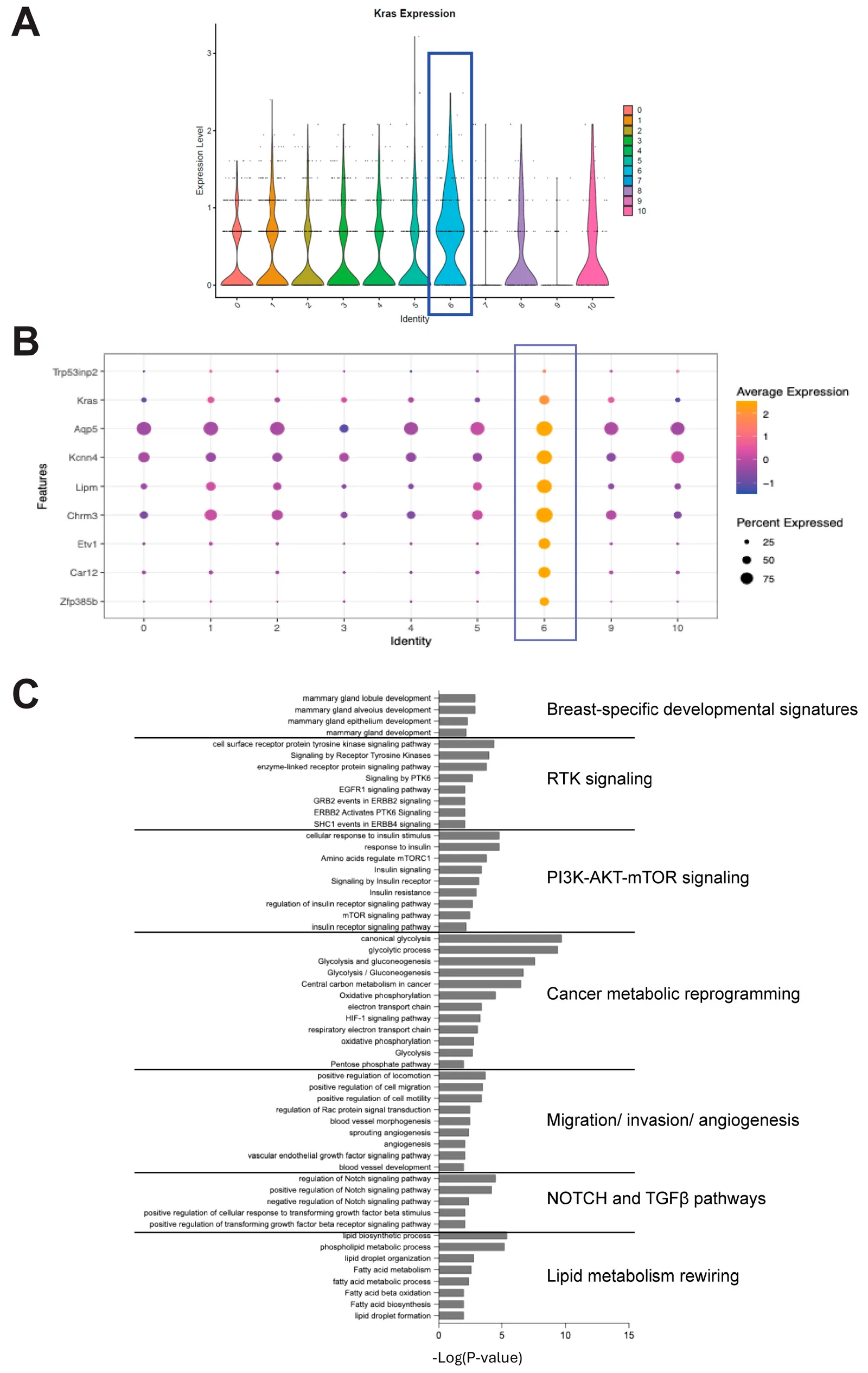
KRAS activation induces an oncogenic-permissive epithelial state characterized by oncogenic signaling and metabolic rewiring. (**A**) Violin plot depicting the distribution of *Kras* expression across GFP-positive cell clusters targeted by the *MMTV-Flp* transgene. Note the elevated expression of *Kras* in the unique cluster 6 of *MMTV-Flp Rosa26*-*GFP FSF*-*Kras^G12D^* experimental females. (**B**) Dot plot showing *Kras* expression and selected marker genes enriched in cluster 6 relative to other epithelial states. (**C**) Gene Ontology (GO) analysis of genes enriched in cluster 6. Selected enriched GO terms were subsequently organized into seven cancer-related functional categories based on their biological functions: breast-specific developmental signatures, receptor tyrosine kinase (RTK) signaling, PI3K-AKT-mTOR signaling, cancer-associated metabolic reprogramming, migration/invasion/angiogenesis, NOTCH and TGFβ signaling, and lipid metabolism rewiring. Bars indicate the statistical significance of GO-term enrichment, expressed as −log(*p* value).

The *MMTV-Flp*-mediated activation of oncogenic KRAS led to the emergence of a distinct epithelial state (cluster 6) adjacent to the central luminal super-cluster. Cells within cluster 6 exhibited elevated expression of *Kras* (Figure 5A), together with enrichment of direct and indirect downstream targets of KRAS signaling. The most prominent marker genes enriched in cluster 6 compared to the other epithelial states within the luminal super-cluster are shown in Figure 5B. GO enrichment analysis of cluster 6 marker genes identified biological processes that were subsequently organized into seven cancer-related functional categories, including mammary developmental programs, RTK and PI3K-AKT-mTOR signaling, metabolic reprogramming, migration/invasion/angiogenesis, NOTCH/TGFβ signaling, and lipid metabolism (Figure 5C). *Etv1* is a canonical effector of KRAS signaling, and the pronounced increase in *Car12*, *Aqp5*, *Kcnn4*, *Lipm*, *Chrm3*, *Trp53inp2*, and *Zfp385b* was likely a consequence of KRAS-driven transcriptional programs that triggered metabolic rewiring, altered ion-transport, and oncogenic stress response that collectively shaped a plastic epithelial state (Figure 5C). The activation of mutant KRAS led to the upregulated expression of stem and progenitor cell markers within the luminal super-cluster, and a higher expression of *Tspan8*, *Procr*, *Gata3*, *Sox9*, *Stat3*, and *Fos* was retained in the emerging cluster 6. While these cells also showed an elevated expression of *Ly6d*, *Lalba*, and *Hey1*, indicative of luminal progenitors with signs of alveolar lineage determination, the combined luminal–basal marker profile and high EMT signature scores signify a pronounced degree of epithelial plasticity (Supplementary Figure S8).

Although no overt tumors were present in the mammary glands of *MMTV-Flp FSF-Kras^G12D^ Rosa26-GFP* females at the time of analysis, the molecular profiles of cluster 6 showed hallmarks of cancer-associated metabolic reprogramming (glycolysis, altered lipid metabolism), activation of oncogenic pathways, and genes that are associated with cancer progression and metastasis (Supplementary Figures S13 and S14). These molecular features may reflect an increased activity of signaling pathways involved in cell proliferation, epithelial lineage determination, and mammary tumorigenesis, including NOTCH, WNT, RTK, and PI3K signaling (Figure 5C). The examination of bulk RNA-seq data from basal-like or claudin-low mammary cancers that developed in mutant KRAS-expressing female mice revealed that five of the cluster 6-defining signature genes (Figure 2 and Figure 5B; i.e., *Car12*, *Lipm*, *Aqp5*, *Chil1*, *and Etv1*) were expressed in established mammary tumors (Supplementary Figure S15). Moreover, three signature genes (*AQP5*, *CHI3L1*, and *KCNN4*) showed significantly higher expression levels in human TNBC cases than in other molecular subtypes (Supplementary Figure S16). The analysis of gene expression values also revealed that, in human tissues, *CA12* (the ortholog of mouse *Car12*) and *ELPOR1* (the ortholog of mouse *5330417C22Rik*) are significantly upregulated in tumors and tumor-adjacent specimens compared with normal breast tissue (Supplementary Figure S17). This analysis also confirmed that *KRAS* expression is generally higher in human breast cancers than in normal controls. Collectively, the enrichment of cancer-associated metabolic and signaling pathways, along with the persistent upregulation of cluster-defining signature genes in mouse mammary tumors and human breast cancers, supports that cluster 6 is a transformation-competent mammary epithelial state.

### 3.5. Trajectory Analyses Associate the Mutant KRAS-Responsive Cellular State with an Alveolar Progenitor, Early-Secretory Differentiation Program

Following the identification of the epithelial lineages and cellular states that were GFP-labeled by the *MMTV-Flp* transgene, we next used Slingshot, TSCAN, and Monocle3 as bioinformatic tools for an inferred ordering of functional groups of luminal epithelial cell types. The primary objective of this analysis was to infer the developmental relationship between cells in the mutant KRAS-responsive cluster and other epithelial cell states. First, we performed a Slingshot analysis on the control and KRAS-expressing epithelial cells to connect clusters along a minimum-spanning tree (Supplementary Figure S18A,B). Although the overall trajectory commonly overlapped, mammary epithelial cells from KRAS-expressing mice exhibited a detour toward clusters 6 and 8, including functionally differentiated cell types, before returning to the sample endpoint (cluster 7). These results suggested that mutant KRAS is associated with alterations in inferred epithelial differentiation trajectories and phenotypic plasticity. Slingshot analysis performed on all epithelial cells by cluster ID revealed the presence of five developmental states that, in part, reflect the previously established groups such as basal/myoepithelial, involution/remodeling luminal progenitors, milk-producing alveolar secretory luminal cells, hormone-responsive luminal cells, and the novel cell population driven by mutant KRAS (Supplementary Figure S18C). These results provide initial confirmation of broad lineage relationships within the GFP-labeled mammary epithelial pool and distinguish clusters that might serve as starting points along the developmental trajectory.

**Figure 6:**
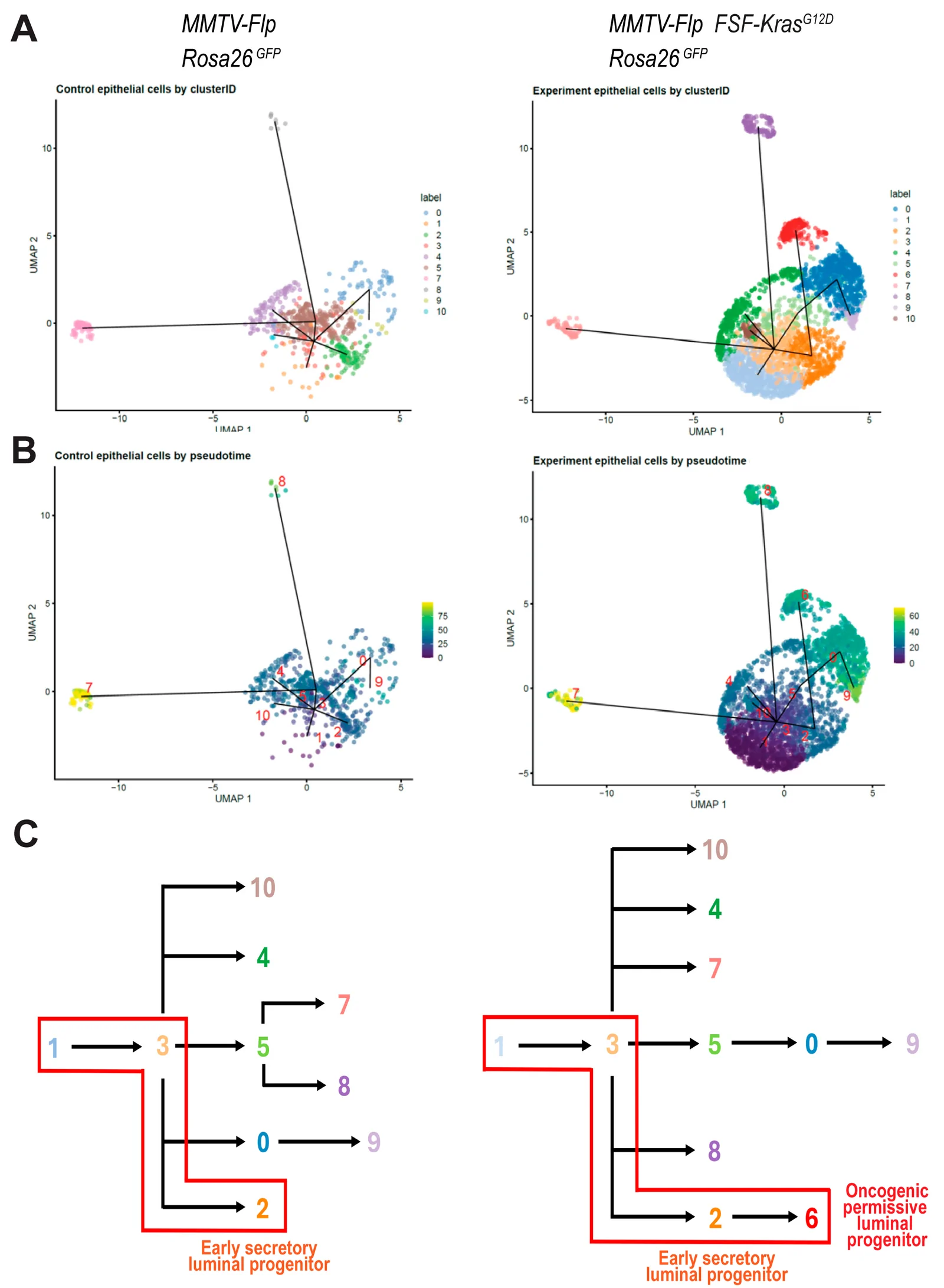
Trajectory inference associates the KRAS-induced transformation-competent state with an alveolar/early-secretory progenitor differentiation path. (**A**) TSCAN-inference relationship among the mammary epithelial cell states from control (**left**) and oncogenic KRAS*^G12D^*-expressing (**right**) mammary glands. (**B**) The same control and KRAS*^G12D^* populations colored according to inferred pseudotime. (**C**) Simplified representations of inferred cluster relationships in control (**left**) and KRAS*^G12D^*-expressing cells (**right**). Red outlines highlight the early-secretory trajectory involving cluster 2. In KRAS*^G12D^*-expressing cells, cluster 2 is connected to the KRAS-associated oncogenic-permissive state (cluster 6). Lines represent computationally inferred transcriptional relationships and do not establish direct lineage ancestry.

To further characterize relationships among the epithelial states, we performed TSCAN trajectory and pseudotime analysis (Figure 6). Consistent with the Slingshot results, TSCAN identified multiple inferred differentiation trajectories and revealed a central branching region involving clusters 1–5 and 10. These states occupied relatively early pseudotime positions, whereas clusters 0, 6, 8, and 9 were positioned later along the inferred trajectories (Figure 6A–C). Notably, in KRAS-expressing epithelial cells, the oncogenic-permissive luminal progenitor state (cluster 6) was connected to the early secretory luminal progenitor state (cluster 2) along the inferred trajectory. This relationship was absent in control cells, in which cluster 6 was not detected. Wave plots of cluster 6-defining signature genes (*Aqp5*, *Car12*, *Etv1*, *Kcnn4*, and *Lipm*) illustrate increasing gene expression along the inferred trajectory toward the cluster 6 endpoint (Supplementary Figure S19). In contrast, expression of the secretory marker *Csn2*, encoding an early milk protein, is higher along the intermediate portion of the path. Although the descriptive reconstruction of pseudotime orientation did not establish lineage ancestry or temporal causality, the findings suggest that oncogenic KRAS is associated with a shift from an early secretory/alveolar-directed differentiation program to an oncogenic-permissive epithelial state. This inferred relationship may also provide a potential link to the accelerated mammary tumor development observed following parity in this model (Supplementary Figure S2). The Monocle3 analysis yielded a pseudotime organization broadly consistent with the Slingshot and TSCAN results, further supporting the inferred relationships among the epithelial states (Supplementary Figure S20). Collectively, the three complementary trajectory-inference approaches supported an association between the KRAS-induced oncogenic-permissive luminal state and an alveolar/early-secretory differentiation program.

## 4. Discussion

Identifying the cellular origins and the molecular mechanisms that drive cellular plasticity and neoplastic transformation is important for understanding the etiology of breast cancer and developing prevention strategies. While examining normal and preneoplastic breast tissues from women with pathogenic mutations in *BRCA1*, Lim et al. [37] observed that luminal progenitors are candidate targets for the initiation of basal-like mammary cancer. More recent work by our team demonstrated that both basal-like and claudin-low triple-negative mammary cancers (i.e., the two most common TNBC subtypes) can originate from the luminal epithelium [22], suggesting that the phenotypic presentation of cancer cells may differ substantially from that of their normal precursor cells [24]. This general notion is supported by computational studies, demonstrating that TNBCs with mesenchymal traits and overall similarities in gene expression profiles may emerge from diverse intrinsic breast cancer subtypes [21]. Our findings on the cellular origins and plasticity of triple-negative mammary cancers were uncovered using experimental models such as the *MMTV-Flp FSF-Kras^G12D^* mice employed in this study, in which the transforming oncogene is targeted to the luminal epithelium in a constitutively active and cell-lineage differentiation-independent manner. Untethering oncogene expression and a GFP reporter from cell-lineage restriction, such as their dependence on the *MMTV* or a milk protein gene promoter, is essential to reveal the innate spectrum of plasticity and objectively trace the developmental paths as well as molecular changes of cells from the earliest stages of transformation to malignant progression [24].

The initial flow cytometry results of this study confirmed that the extended long terminal repeat (LTR) of the *MMTV* is primarily expressed in the luminal epithelium of the mammary gland [38]. The subsequent investigation of the transcriptomes of single GFP-labeled mammary epithelial cells revealed that the *MMTV*-driven expression of Flp recombinase facilitates the perpetual activation of responder genes (*Rosa26-GFP* and *FSF-Kras^G12D^*) in 11 transcriptionally distinct epithelial cell states (i.e., 10 clusters shared with controls and one unique state initiated by mutant KRAS) that encompass six functional groups. The heterogeneity of cellular populations within the small subset of GFP-labeled epithelial cells was higher than anticipated and included luminal epithelial clusters previously described by Bach et al. [39] and Kumar et al. [40]. More importantly, mutant KRAS activation in the luminal epithelium was associated with the emergence of a distinct, epithelial state (cluster 6), characterized by cellular plasticity (i.e., acquisition of basal and mesenchymal features) and extensive rewiring of signaling and metabolic programs. Three computational trajectory-inference approaches positioned cluster 6 along an alveolar/early-secretory differentiation trajectory. Although this inferred association with an alveolar-directed progenitor trajectory was somewhat unexpected, it provides a nexus between KRAS-induced epithelial plasticity and accelerated tumor development in parous females. The results are similar to earlier observations showing that tumors in *MMTV-ERBB2/neu* transgenic females frequently originate from parity-induced mammary epithelial cells (PI-MECs) or transiently differentiating cells within lobular units of virgin mice [41]. In aggregate, these findings may support the previously stated notion that a subset of ductal carcinoma in situ and invasive adenocarcinomas in women may originate within terminal duct lobular units (TDLUs), which are the histological equivalents to lobular units in mice [42,43].

Although the direct contribution of cluster 6 to mammary tumor formation remains to be experimentally confirmed, we characterize this cell population as a transformation-competent mammary epithelial state because it emerged exclusively in mutant KRAS-expressing glands and showed enrichment of oncogenic signaling pathways and features of increased cellular plasticity. Furthermore, we found that five of the cluster-defining signature genes were highly expressed in triple-negative mammary cancers from *MMTV-Flp FSF-Kras^G12D^* transgenic mice. Three signature genes (*CHI3L1*, *AQP5*, and *KCNN4*) were significantly upregulated in human breast cancers, with a predominant presence in the TNBC subtype. These genes have been reported to mediate biologically relevant functions in mammary cancer initiation and progression. A recent study by Taifour et al. [44] showed that the expression of CHI3L1 in cancer cells is driven by inflammatory cytokine signaling through STAT3, and that the deletion of the gene delays tumor onset by suppressing an anti-tumor immune response. Compared with normal breast epithelial cells, AQP5 levels are significantly higher in the triple-negative MDA-MB-231 cell line, where this protein promotes proliferation, cell survival, and EMT by activating WNT signaling [45]. Unlike CHI3L1, the roles of AQP5 and KCNN4 in mammary cancer initiation remain to be established, but KCNN4 expression has been shown to contribute to TNBC chemoresistance and has been mechanistically linked to RAS-MAPK and PI3K signaling [46].

Beyond the identification of the luminal transcriptionally distinct epithelial state with signature genes that are relevant to TNBC, our collective findings are in agreement with previous observations that increased RAS signaling promotes stemness and induces mesenchymal features in cultured human breast epithelial cells [47]. In support of this concept, the overexpression of *Twist1* in the mammary gland epithelium accelerated the formation of KRAS-induced tumors [48], demonstrating that the acquisition of cellular plasticity is an early event during neoplastic transformation. Single-cell transcriptomic approaches like PIP-seq offer new opportunities to delineate the earliest events in mammary cancer initiation, and future studies may identify shared and distinct features of oncogenic-permissive states within the normal epithelium induced by diverse oncogenes. There is also a clear need to apply single-cell transcriptomics to healthy breast tissues from breast cancer patients, particularly those with genetic predisposition (e.g., germline mutations in TP53 and BRCA1/2), which would enable comparisons with genetic models and validate their relevance to human disease.

Limitations of the study. Although a Flp recombinase-based reporter gene (*Rosa26^FSF-GFP^*) was used for the targeted isolation and sequencing of GFP-positive mammary epithelial cells from controls and experimental females expressing mutant KRAS, the direct contribution of cluster 6 as the primary precursor for mammary tumor formation in response to mutant KRAS remains to be established using additional lineage-tracing methods. Moreover, GFP reporter activation indicates recombination at the *Rosa26* reporter locus but does not directly verify recombination at the *Kras* locus or expression of the mutant allele in every sequenced cell. Second, because of the mosaic expression of the *MMTV-Flp* transgene, the generation of PIP-seq libraries required the pooling of GFP-positive mammary epithelial cells from multiple females, particularly in the control group. Therefore, the data establish library-level recurrence rather than animal-level reproducibility and do not exclude all technical effects, such as those caused by enzymatic tissue dissociation and cell sorting. Third, the computational trajectory reconstruction describes transcriptional relationships between cellular states but does not infer a factual lineage topology. Nonetheless, the projected pseudotime of alveolar progenitors as precursors for the mutant KRAS-induced mammary epithelial state is consistent with the tumor-promoting effects of parity in this animal model.

## 5. Conclusions

The collective findings of this study demonstrate that activation of oncogenic KRAS within the luminal epithelium is associated with the emergence of a distinct epithelial state. Trajectory analyses consistently positioned this oncogenic-permissive state along an alveolar progenitor differentiation program. The state was characterized by increased cellular plasticity and extensive rewiring of major signaling and metabolic programs. Moreover, the upregulated expression of selected signature genes defining the transcriptionally distinct luminal cell population is retained in malignant triple-negative mammary tumors. These results support the concept that oncogene-driven disruption of normal epithelial differentiation is an early event during mammary tumorigenesis and identify molecular programs that warrant further investigation as potential targets for cancer prevention.

## Data Availability

The RNA-seq data files generated in this study have been deposited in the Gene Expression Omnibus (GEO) database under accession code GSE345742.

## Supplementary Materials

The following supporting information can be downloaded at: https://www.mdpi.com/article/10.3390/cancers18213483/s1, Figure S1. Isolation and single-cell transcriptomic analysis of GFP-labeled mammary epithelial cells from MMTV-Flp Rosa26-GFP FSF-KrasG12D females. (A). Schematic illustration of experimental and control mouse models. (B). Sequential workflow outlining mammary epithelial cell isolation for downstream single-cell RNA-sequencing. Illustrations were generated using BioRender.com; Figure S2. Parity accelerates mammary tumor onset in MMTV-Flp FSFKras G12D mice. Kaplan-Meier plot comparing the mammary tumor-free survival between nulliparous and parous MMTV-Flp FSF-KrasG12D females. Statistical significance between the two survival curves was calculated using the log-rank (Mantel-Cox) test (*p* < 0.0001); Figure S3. Initial analysis of the scRNA-seq datasets identified 15 transcriptionally distinct clusters among GFP-sorted cells from the mammary glands of experimental and control mice. (A). UMAP representation of the combined control (MMTV-Flp Rosa26-GFP) and experimental (MMTV-Flp Rosa26-GFP FSF-KrasG12D) datasets, identifying 15 transcriptionally distinct cell clusters. (B). Distribution of cells across the identified clusters. (C). UMAP projections of GFP-sorted cells from control females (left) and KRASG12D-expressing mice (right), revealing a KRAS-associated population (initial cluster 7) that was subsequently designated cluster 6 following exclusion of non-epithelial populations and de novo re-clustering of the epithelial-cell dataset; Figure S4. Cellular identity and GFP expression across the 15 cellular populations originally distinguished during preliminary clustering. (A). UMAP representation of the module scores of defined gene sets indicative of basal and luminal epithelial cell identity within the combined datasets of MMTV-Flp Rosa26-GFP females with and without the conditional FSF-KrasG12D allele. (B). UMAP showing the transcriptional activation of the Gfp reporter transcripts across the 15 initially identified cell types; Figure S5. Expression of lineage-specific markers across the 15 initially identified cell populations. Dot plots showing the gene expression of marker genes associated with epithelial, mesenchymal, endothelial, fibroblasts, and immune cells across the individual cell populations. Dot color represents average gene expression; dot size represents the percentage of cells expressing each gene; Figure S6. Exclusion of immune cells for the analysis of GFP-expressing mammary epithelial cells in response to the activation of the *MMTV-Flp* transgene. (A). Violin plots showing expression of immune and endothelial cell markers (*Cd3e*, *Cd19*, *Pecam1*, and *Cdh5*) across the major and minor cell populations isolated by GFP-based fluorescenceactivated cell sorting from the mammary glands of *MMTV-Flp Rosa26*-*GFP* females with and without the conditional *FSF-KrasG12D* allele. (B). Violin plot highlighting the expression of *Ptprc* (CD45) across the major and minor cell populations identified by scRNA-seq. *Ptprc* expression was used as an exclusion criterion to remove immune cells from the subsequent analysis of mammary epithelial cell clusters; Figure S7. Minor cluster 7 exhibits elevated expression of genes characteristic of basal epithelial cells. (A). Violin plot depicting the distribution of Gfp expression across cell clusters targeted by the MMTV-Flp transgene. (B). UMAP plots of the basal mammary epithelial cell-associated genes Krt5, Krt14, Acta2, Myc, and Id4, the arrow points to cluster 7, which has luminal features but contains cells that show elevated levels of basal epithelial markers. (C). UMAP representation of the module scores of defined gene sets indicative of basal and luminal epithelial cell identities following exclusion of immune cells; Figure S8. Emergence of a unique cellular population (cluster 6) exclusively identified in oncogenic KRAS-expressing samples. Uniform Manifold Approximation and Projection (UMAP) plots of eleven transcriptionally distinct mammary epithelial cell states (clusters 0–10) in two independent datasets from MMTVFlp Rosa26-GFP control females (upper panels) and two independent datasets from MMTV-Flp Rosa26-GFP FSF-KrasG12D experimental females (lower panels); Figure S9. The unique KRAS-associated cellular state (cluster 6) is consistently and exclusively identified across independent experimental libraries. (A). UMAP projections of mammary epithelial cells from two independently processed pooled control libraries (Ctrl. 1 and Ctrl. 2; blue shades) and two independently processed pooled KRASexpressing experimental libraries (Exp. 1 and Exp. 2; red/orange shades). Cells are shown together (top) and separately by genotype (bottom). (B). Heatmap showing the percentage of epithelial cells assigned to each cluster within each library (top), and stacked bar plot showing the relative contribution of each library to each cluster (bottom). Cluster 6 was detected in both experimental libraries and was not present in either control library. These observations support recurrence across experimental libraries but do not establish reproducibility among individual mice or exclude all technical effects; Figure S10. Gene Ontology (GO) enrichment analysis of biological processes across the 11 mammary epithelial cell states. Gene Ontology (GO) analysis of molecular pathways enriched across all 11 cellular states in comparison to the KRAS-induced cluster 6 (boxed). Box colors indicate enrichment as −log10(Pvalue); Figure S11. Unique presence of the oncogenic-permissive functional group (cluster 6, green) in experimental females expressing mutant KRAS. (A). UMAP plot of GFP-expressing epithelial cell clusters within functional groups as defined by the top 20 most differentially expressed genes in MMTV-Flp Rosa26-GFP control females (left) and MMTV-Flp Rosa26-GFP FSF-KrasG12D experimental females (right). (B). Relative number of cells within each functional group across two independently processed libraries from MMTV-Flp Rosa26-GFP control females (Ctrl. 1/2) and two independently processed libraries from MMTV-Flp Rosa26-GFP FSF-KrasG12D experimental females (Exp. 1/2); Figure S12. The luminal–basal marker profile and high EMT signature scores suggest a pronounced degree of epithelial plasticity in mammary epithelial cells expressing oncogenic KRAS. UMAP representation of the CK8/CK14 dual-positivity module score (A) and the epithelial-tomesenchymal (EMT) module score (B) in GFP-positive mammary epithelial cells from MMTVFlp Rosa26-GFP control females (left) and MMTV-Flp Rosa26-GFP FSF-KrasG12D experimental females (right); Figure S13. The novel epithelial state (cluster 6) in response to oncogenic KRAS activation is characterized by enrichment of genes associated with mammary development and metabolic rewiring. (A). Dot plot illustrating the expression profiles of a 10-gene signature associated with mammary gland development across all 11 epithelial cell states identified in GFP-positive mammary epithelial cells isolated from MMTV-Flp Rosa26-GFP FSF-KrasG12D females. (B). Dot plot depicting the expression profiles of genes associated with lipid metabolism across all 11 epithelial cell states. Dot color represents average gene expression; dot size represents the percentage of cells expressing each gene; Figure S14. Mammary epithelial cells induced by mutant KRAS in cluster 6 exhibit elevated expression of genes associated with oncogenic signaling and tumor progression. (A). Dot plot visualizing the expression profiles of genes associated with breast cancer and oncogenic signaling pathways across all 11 epithelial cell states identified among GFP-positive mammary epithelial cells isolated from MMTV-Flp Rosa26-GFP FSF-KrasG12D females. (B). Dot plot displaying the expression profiles of genes associated with metastasis and tumor progression across all 11 epithelial cell states. Dot color represents average gene expression; dot size represents the percentage of cells expressing each gene; Figure S15. Elevated expression of selected signature genes defining the novel transformation-competent mammary epithelial state (cluster 6) in mutant KRAS-induced basal-like and claudin-low mammary tumors. (A). RNA-seq coverage tracks showing normalized expression profiles of selected genes that discriminate five basal epithelial-like from five claudin-low (mesenchymal-like) mammary tumors and two normal mammary glands. (B). RNA-seq coverage tracks showing elevated expression of five cluster 6-defining genes in triplenegative mammary cancers that originated in MMTV-Flp FSF-KrasG12D females. The plots illustrate the transcript coverage across individual exons; Figure S16. Upregulated expression of selected genes defining the unique transformation-competent mammary epithelial state (cluster 6) across human breast cancer subtypes. Box plots illustrate the significantly elevated expression of AQP5, CHI3L1, and KCNN4 across five human molecular breast cancer subtypes (data source TCGA, Breast Cancer Gene-Expression Miner v5.3); Figure S17. Elevated expression of selected genes defining the KRAS-induced transformation-competent mammary epithelial state (cluster 6) in human breast tissue and tumors. Box plots illustrating significantly elevated expression levels of CA12 and ELAPOR1 in tumoradjacent normal breast tissue and breast cancers relative to normal breast tissue (data source: TCGA, Breast Cancer Gene-Expression Miner v5.3); Figure S18. Slingshot-inferred differentiation trajectories and pseudotime ordering of mammary epithelial cell states in control and KRAS-expressing mice. Slingshot lineage and pseudotime analysis characterizing major mammary epithelial differentiation trajectories and assessing KRAS-associated differences across the 11 epithelial cell clusters identified in MMTV-Flp Rosa26-GFP control females (A) and MMTV-Flp Rosa26-GFP FSF-KrasG12D experimental females (B). (C). UMAP visualization of the combined control and experimental samples showing five inferred trajectories across the GFP-positive epithelial cell populations. Outlier cells were excluded prior to trajectory analysis. Black lines represent inferred differentiation lineages, and cells are colored by their established cluster identity; Figure S19. Expression dynamics of genes defining the unique oncogenicpermissive mammary epithelial state (cluster 6) in KRAS-expressing mammary epithelial cells. Gene expression wave plots for individual genes defining the unique KRAS-induced cellular state along the reconstructed experimental TSCAN path ending in cluster 6. Expression was summarized in 20 equal-width pseudotime bins, with point size indicating the number of cells per bin. Expression of the cluster 6-defining genes Aqp5, Car12, Etv1, Kcnn4, and Lipm (blue boxes) increases toward the cluster 6 end, whereas the secretory alveolar marker Csn2 and Lalba are higher in the intermediate portion of the path. Following the trajectory of cluster 6-defining genes, Kras expression increases towards the cluster 6 endpoint; Figure S20. Monocle3-inferred differentiation trajectories and pseudotime ordering of GFP-positive mammary epithelial cells from control and KRAS-expressing mice. (A). UMAP representation of the 11 mammary epithelial states according to cluster identity. (B). Monocle3-inferred trajectory colored according to pseudotime progression. (C). Distribution of epithelial cell states along inferred pseudotime, visualizing their relative positions within the reconstructed trajectory; Table S1. PCR primers to genotype transgenes and genetically engineered alleles; Table S2. List of primary antibodies used for flow cytometric analysis; Table S3. List of primary antibodies used for lineage depletion; Table S4. PIP-seq library index and adapter sequence summary for sequencing.

## Abbreviations

BRCA1	Breast Cancer 1, DNA repair associated.
CD3E	CD3 epsilon subunit of T-cell receptor complex
CD24	Cluster of Differentiation 24
CD29	Integrin Beta 1, ITGB1
CD44	Homing cell adhesion molecule, HCAM
CD45	Protein Tyrosine Phosphatase Receptor Type C, PTPRC
CD49f	Integrin alpha 6, ITGA6
CD61	Integrin beta 3, ITGB3
CDH1	Cadherin 1, E-cadherin
CDH2	Cadherin 2, N-cadherin
CK14	Cytokeratin 14, KRT14
CK8	Cytokeratin 8 (KRT8)
Cre	Cre recombinase from the P1 bacteriophage
CLBC	Claudin-Low Breast Cancer
DMEM	Dulbecco’s Modified Eagle Medium
EMT	epithelial-to-mesenchymal transition
EpCAM	epithelial cell adhesion molecule
ERBB2	ErbB2 receptor tyrosine kinase 2, HER2, neu
ETV1/Etv1	ETS variant transcription factor 1
FACS	fluorescence-activated cell sorting
Flp	Site-specific DNA recombinase from *Saccharomyces cerevisiae*
FSC	Forward scatter
GFP	Green fluorescent protein
HER2	Human epidermal growth factor receptor 2, ERBB2
HRAS	HRAS proto-oncogene, GTPase
IDC	Invasive ductal carcinoma
IgA	Immunoglobulin A
IHC	Immunohistochemistry
ILC	Invasive lobular carcinoma
KRAS	KRAS proto-oncogene, GTPase
LTR	Long terminal repeat, regulatory region
MAPK	Mitogen-activated protein kinase
MEC	Mammary epithelial cell
MMTV	Mouse mammary tumor virus
mTOR	Mammalian target of rapamycin
PCA	Principal component analysis
PE/Cy7	Phycoerythrin/Cyanine 7 tandem fluorophore
PECAM1	Platelet and endothelial cell adhesion molecule 1
PI3K	Phosphoinositide 3-kinase
PIP-seq	Particle-templated instant partition sequencing
RNA-seq	RNA sequencing
Rosa26	Gt(ROSA)26Sor, mouse genomic locus on chromosome 6
RTK	Receptor tyrosine kinase
scRNA-seq	Single-cell RNA-sequencing
SSC	Side scatter
TetO	Tetracycline-responsive operator
TGF	Transforming growth factor
TNBC	Triple-negative breast cancer
TSG101	Tumor susceptibility gene 101
tTA	Tetracycline-controlled transactivator, Tet-Off
UMAP	Uniform Manifold Approximation and Projection
VIM	Vimentin, type III intermediate filament protein
