# Supplementary material for: Mutant KRAS Reprograms Luminal Progenitor Differentiation to Establish a Novel Transformation-Competent Mammary Epithelial Cell State
Source: Cancers (Basel). 2026 Oct 28;18(21):3483. doi: 10.3390/cancers18213483 (PMC13761582; doi:10.3390/cancers18213483)
Supplement: Supplementary file 1 [file cancers-18-03483-s001.zip › cancers-4588824-supplementary.pdf]

Supplemental information

Mutant KRAS reprograms luminal progenitor differentiation to establish a novel transformation-competent mammary epithelial cell state

Madison N. Wicker, Hye Kyung Lee, Kerry Vistisen and Kay-Uwe Wagner

Supplemental Figures S1-S20

Supplementary Tables S1-S4

Addendum: Flow cytometric images, gating strategy

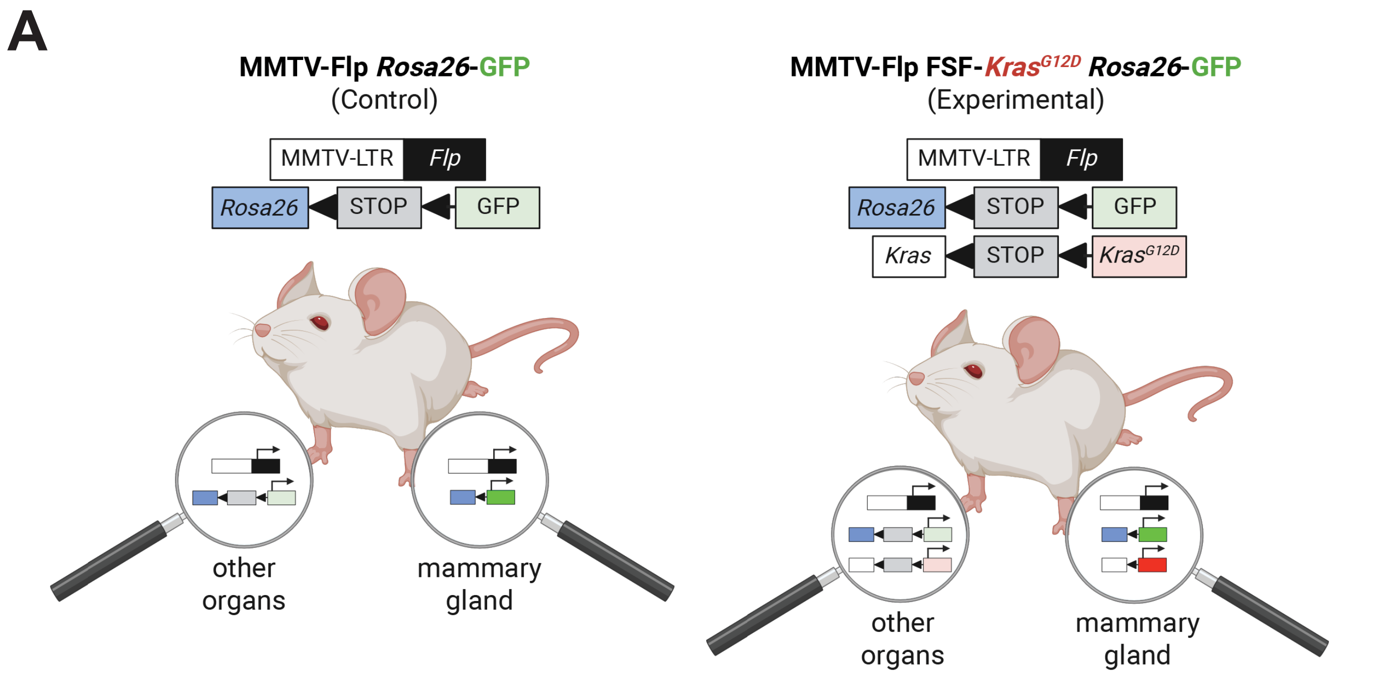
A
MMTV-Flp Rosa26-GFP
(Control)
MMTV-LTR
Flp
Rosa26
STOP
GFP
other organs
mammary gland
MMTV-Flp FSF-KrasG12D Rosa26-GFP
(Experimental)
Kras
STOP
KrasG12D
other organs
mammary gland

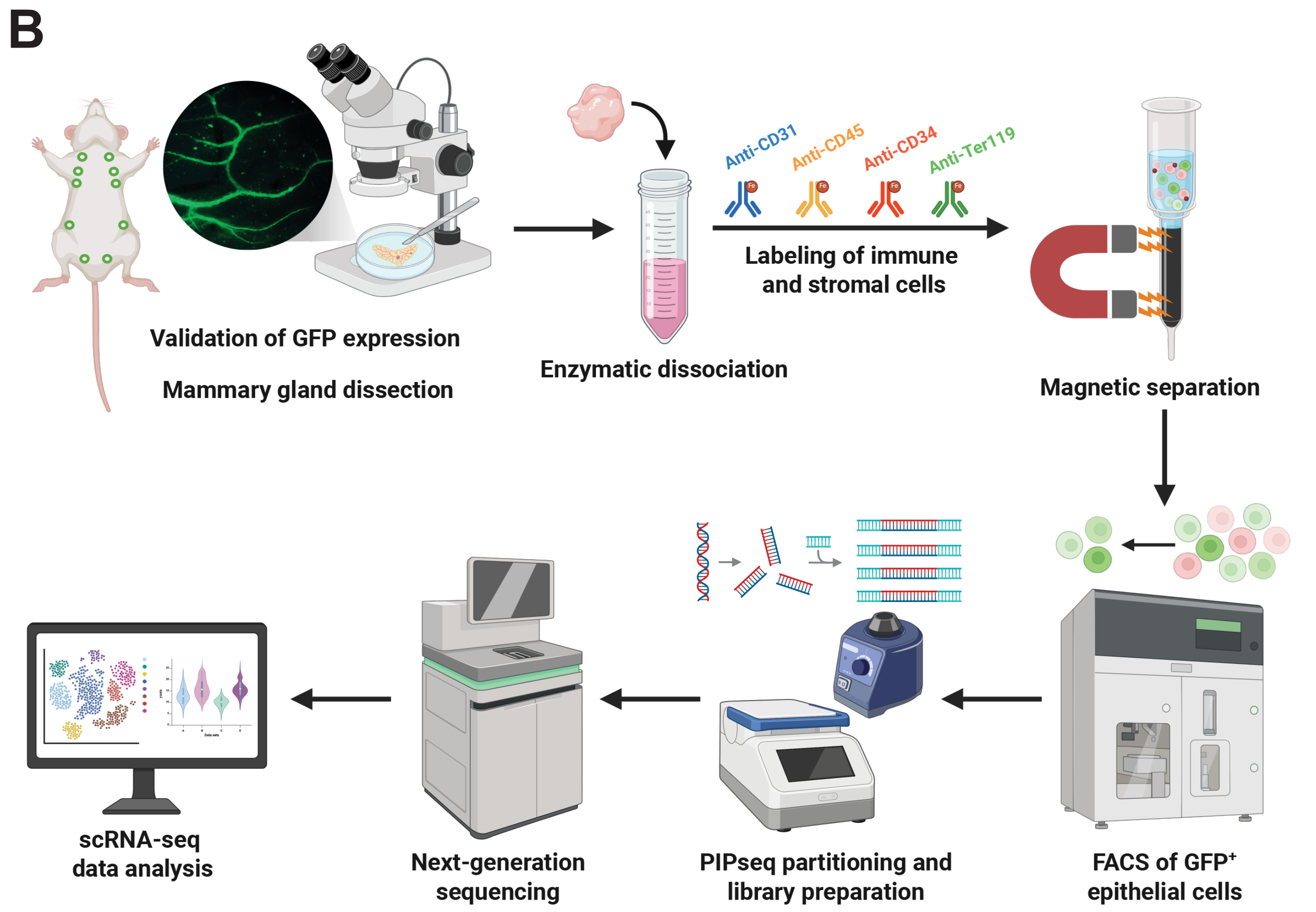
B
Validation of GFP expression
Mammary gland dissection
Enzymatic dissociation
Anti-CD31
Anti-CD45
Anti-CD34
Anti-Ter119
Labeling of immune and stromal cells
Magnetic separation
FACS of GFP+ epithelial cells
PIPseq partitioning and library preparation
Next-generation sequencing
scRNA-seq data analysis

Supplemental Figure S1. Isolation and single-cell transcriptomic analysis of GFP-labeled mammary epithelial cells from *MMTV-Flp Rosa26-GFP FSF-Kras*^{G12D} females.

A. Schematic illustration of experimental and control mouse models.

B. Sequential workflow outlining mammary epithelial cell isolation for downstream single-cell RNA-sequencing. Illustrations were generated using BioRender.com.

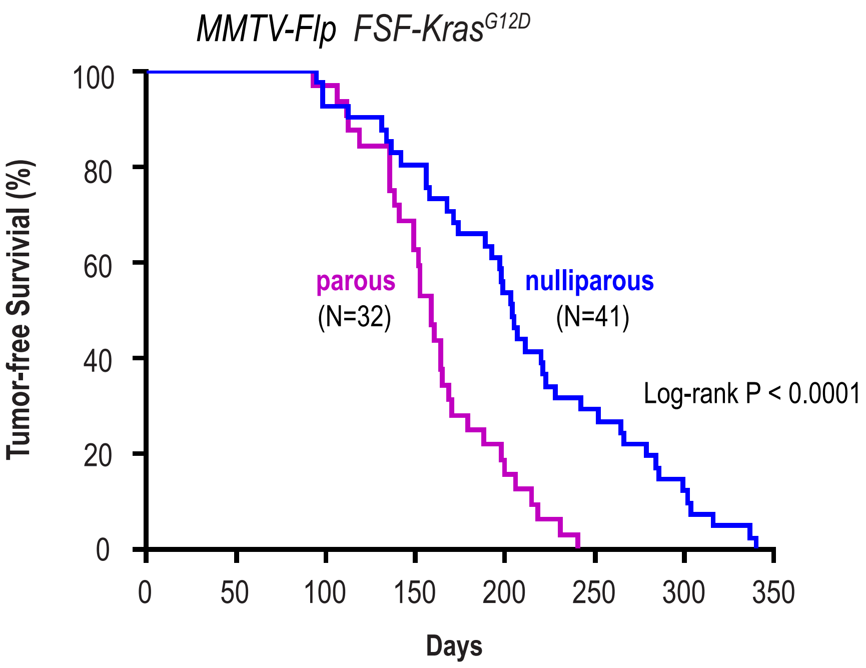
MMTV-Flp FSF-Kras^{G12D}
Tumor-free Survivial (%)
100
80
60
40
20
0
parous
(N=32)
nulliparous
(N=41)
Log-rank P < 0.0001
0
50
100
150
200
250
300
350
Days

Supplemental Figure S2. Parity accelerates mammary tumor onset in *MMTV-Flp FSF-Kras*^{G12D} mice.

Kaplan-Meier plot comparing the mammary tumor-free survival between nulliparous and parous *MMTV-Flp FSF-Kras*^{G12D} females. Statistical significance between the two survival curves was calculated using the log-rank (Mantel-Cox) test ($P < 0.0001$).

Supplemental Fig. S3 Wicker et al.

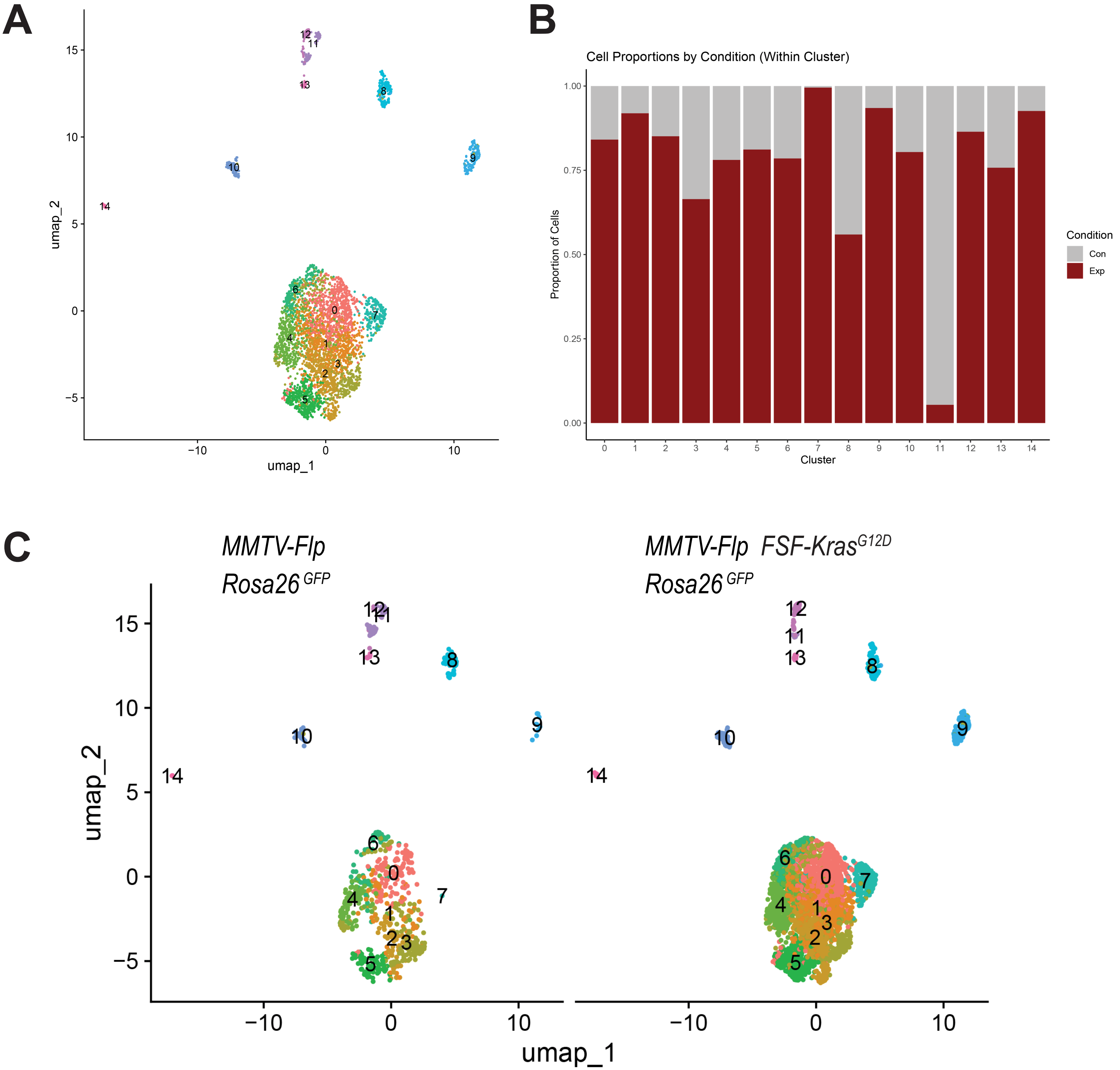

Supplemental Figure S3. Initial analysis of the scRNA-seq datasets identified 15 transcriptionally distinct clusters among GFP-sorted cells from the mammary glands of experimental and control mice.

A. UMAP representation of the combined control (*MMTV-Flp Rosa26-GFP*) and experimental (*MMTV-Flp Rosa26-GFP FSF-Kras*^{G12D}) datasets, identifying 15 transcriptionally distinct cell clusters.

B. Distribution of cells across the identified clusters.

C. UMAP projections of GFP-sorted cells from control females (left) and KRAS^{G12D}-expressing mice (right), revealing a KRAS-associated population (initial cluster 7) that was subsequently designated cluster 6 following exclusion of non-epithelial populations and *de novo* re-clustering of the epithelial-cell dataset.

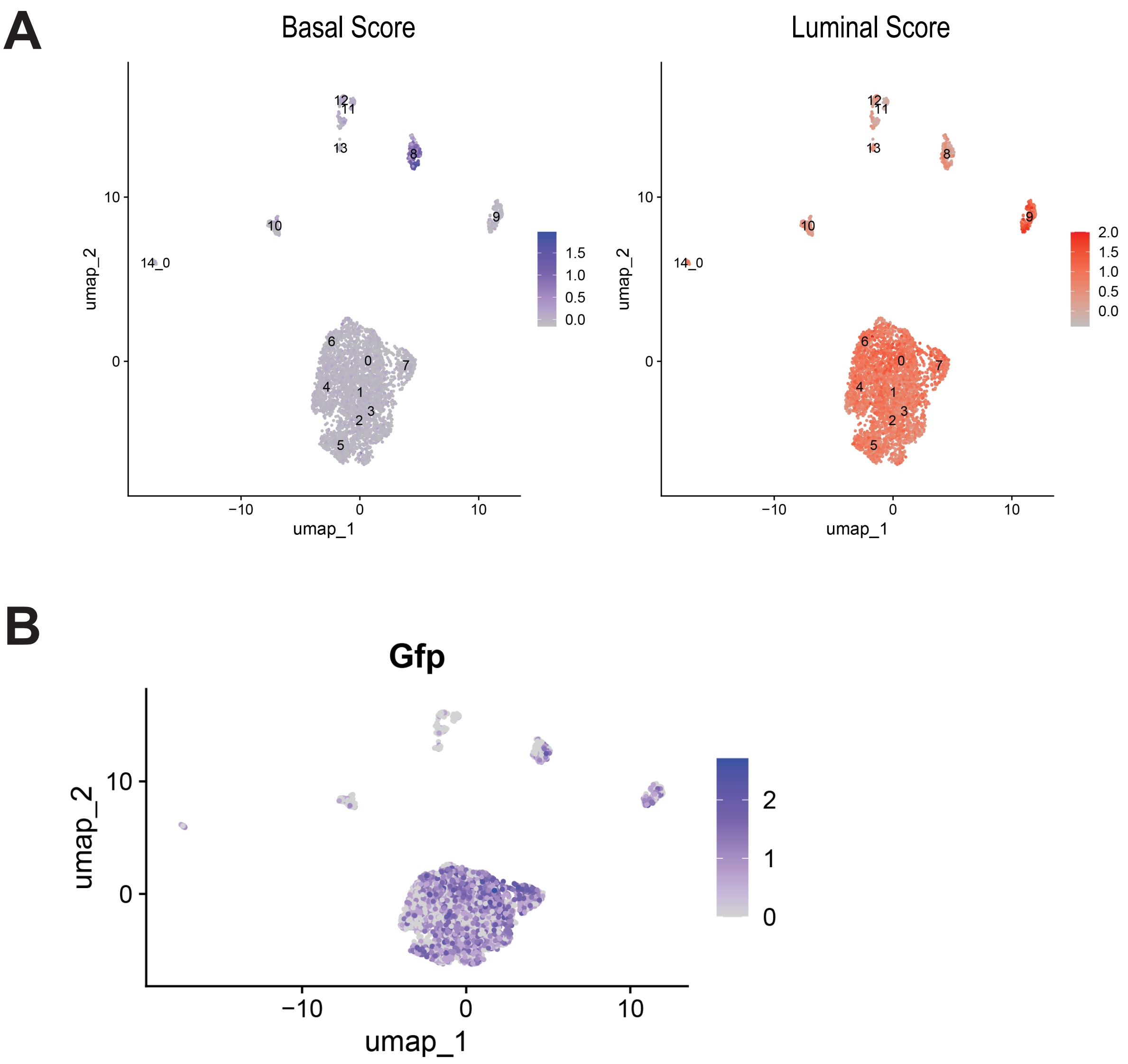
A
Basal Score
Luminal Score
umap_2
umap_1
14_0
10
9
8
12
11
13
6
0
7
4
1
3
2
5
−10
0
10
1.5
1.0
0.5
0.0
2.0
B
Gfp
2
1
0

Supplemental Figure S4. Cellular identity and GFP expression across the 15 cellular populations originally distinguished during preliminary clustering.

A. UMAP representation of the module scores of defined gene sets indicative of basal and luminal epithelial cell identity within the combined datasets of *MMTV-Flp Rosa26-GFP* females with and without the conditional *FSF-Kras*^{G12D} allele.

B. UMAP showing the transcriptional activation of the *Gfp* reporter transcripts across the 15 initially identified cell types.

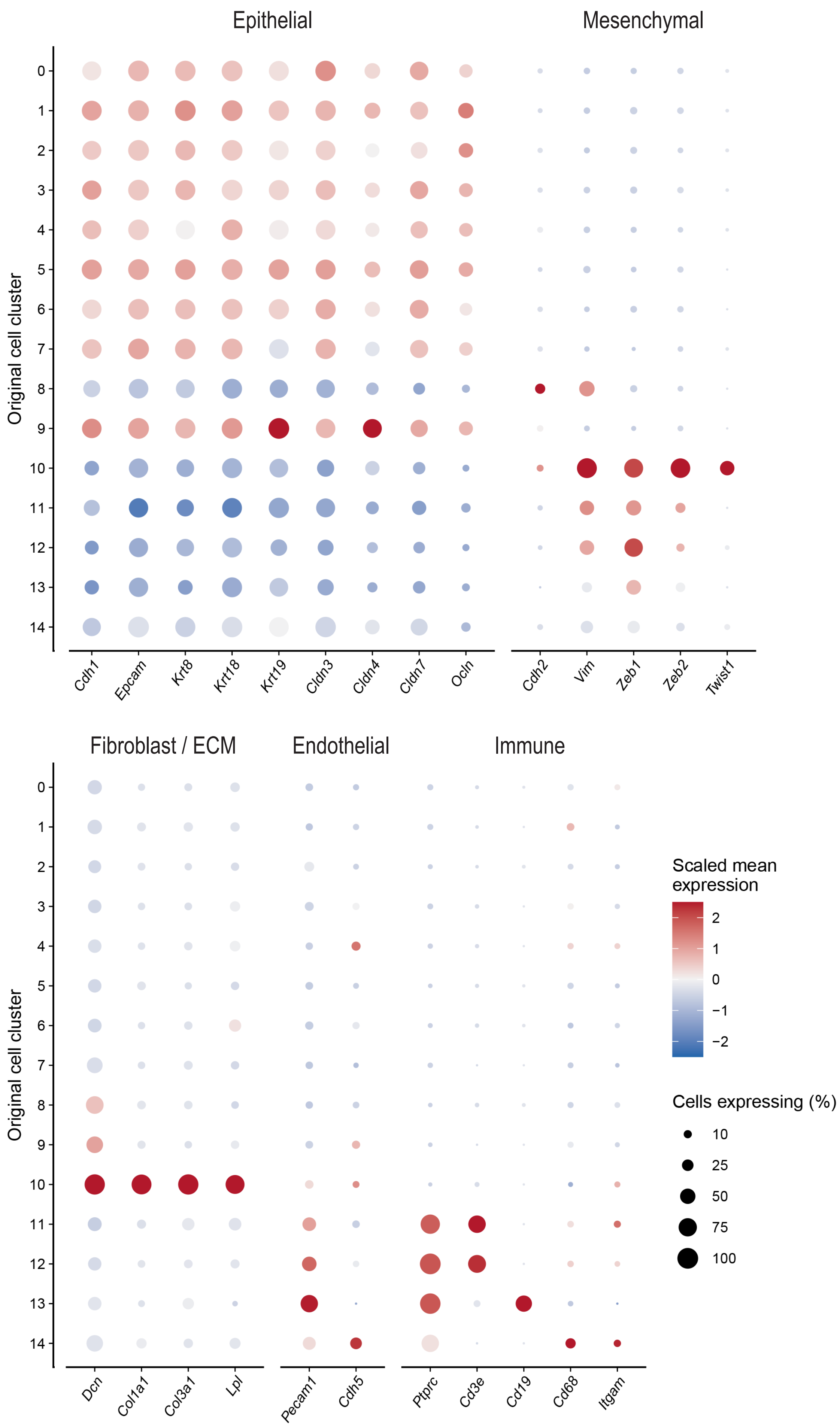
Epithelial
Mesenchymal
Original cell cluster
0
1
2
3
4
5
6
7
8
9
10
11
12
13
14
Cdh1
Epcam
Krt8
Krt18
Krt19
Cldn3
Cldn4
Cldn7
Ocln
Cdh2
Vim
Zeb1
Zeb2
Twist1
Fibroblast / ECM
Endothelial
Immune
Dcn
Col1a1
Col3a1
Lpl
Pecam1
Cdh5
Ptprc
Cd3e
Cd19
Cd68
Itgam
Scaled mean expression
2
1
0
−1
−2
Cells expressing (%)
10
25
50
75
100

Supplemental Figure S5. Expression of lineage-specific markers across the 15 initially identified cell populations.

Dot plots showing the gene expression of marker genes associated with epithelial, mesenchymal, endothelial, fibroblasts, and immune cells across the individual cell populations. Dot color represents average gene expression; dot size represents the percentage of cells expressing each gene.

Supplemental Fig. S6

Wicker et al.

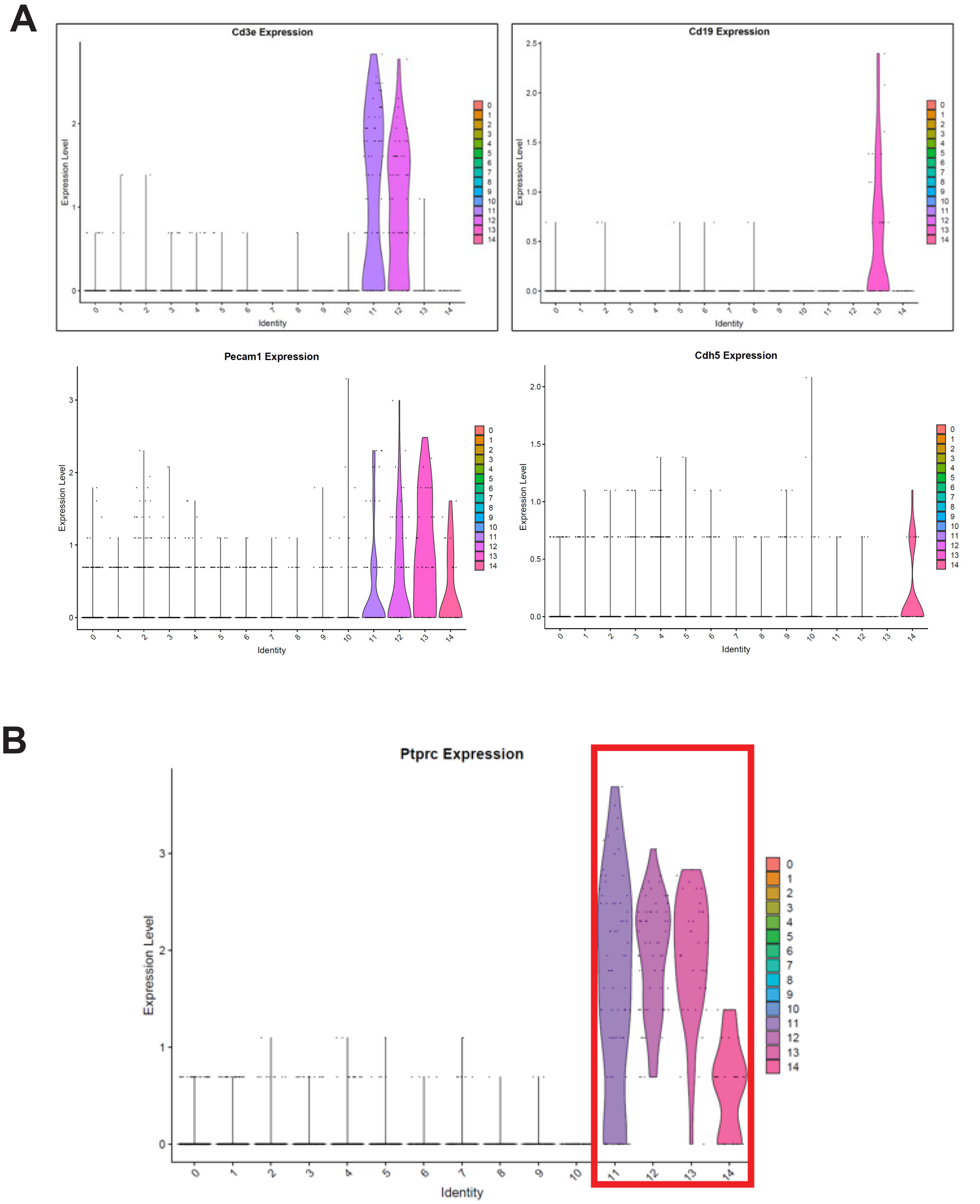

Supplemental Figure S6. Exclusion of immune cells for the analysis of GFP-expressing mammary epithelial cells in response to the activation of the *MMTV-Flp* transgene.

A. Violin plots showing expression of immune and endothelial cell markers (*Cd3e*, *Cd19*, *Pecam1*, and *Cdh5*) across the major and minor cell populations isolated by GFP-based fluorescence-activated cell sorting from the mammary glands of *MMTV-Flp Rosa26-GFP* females with and without the conditional *FSF-Kras*^{G12D} allele.

B. Violin plot highlighting the expression of *Ptprc* (CD45) across the major and minor cell populations identified by scRNA-seq. *Ptprc* expression was used as an exclusion criterion to remove immune cells from the subsequent analysis of mammary epithelial cell clusters.

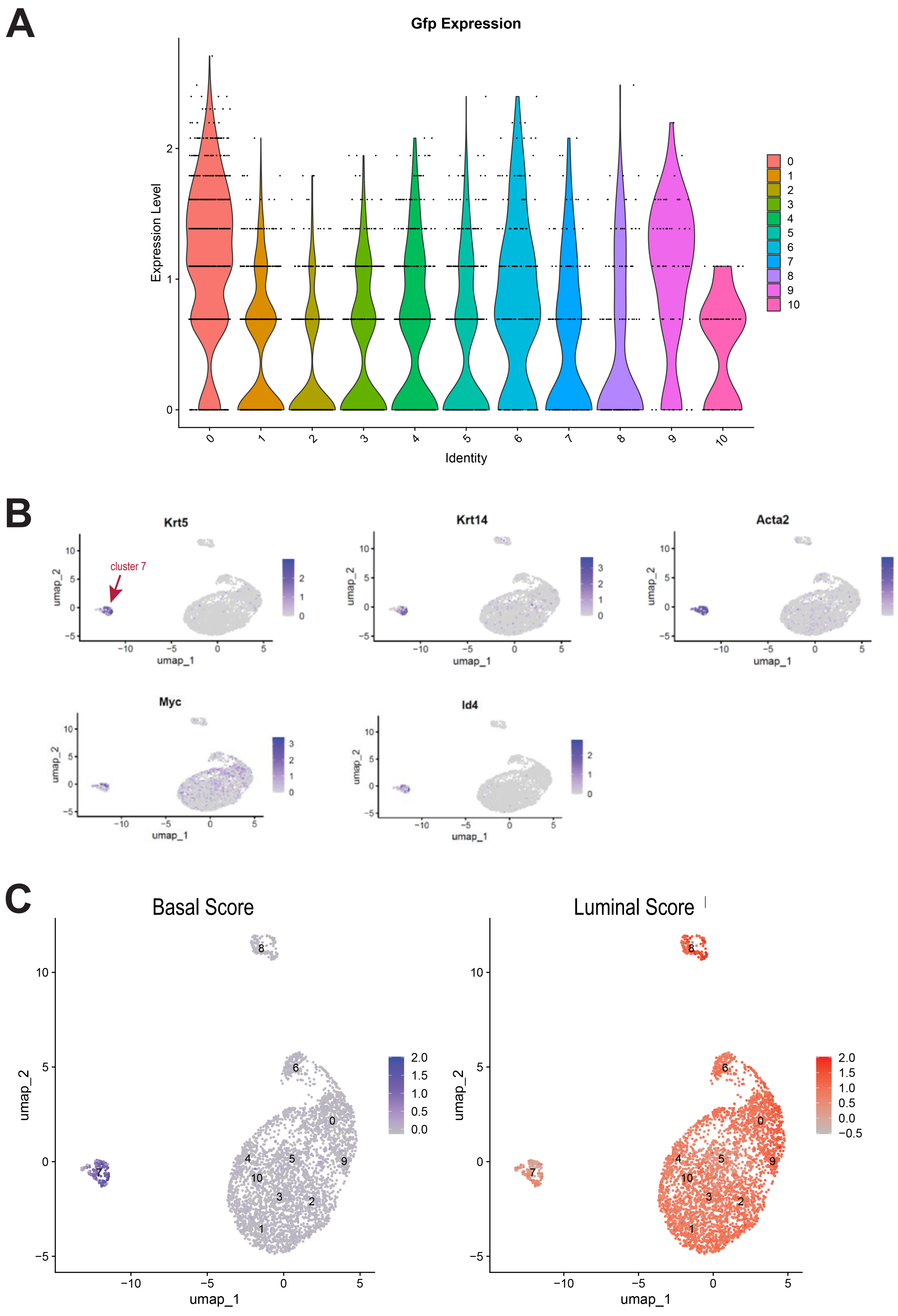
A
Gfp Expression
Expression Level
Identity
B
Krt5
Krt14
Acta2
Myc
Id4
cluster 7
umap_1
umap_2
C
Basal Score
Luminal Score
umap_1
umap_2

Supplemental Figure S7. Minor cluster 7 exhibits elevated expression of genes characteristic of basal epithelial cells.

A. Violin plot depicting the distribution of *Gfp* expression across cell clusters targeted by the *MMTV-Flp* transgene.

B. UMAP plots of the basal mammary epithelial cell-associated genes *Krt5*, *Krt14*, *Acta2*, *Myc*, and *Id4*, the arrow points to cluster 7, which has luminal features but contains cells that show elevated levels of basal epithelial markers.

C. UMAP representation of the module scores of defined gene sets indicative of basal and luminal epithelial cell identities following exclusion of immune cells.

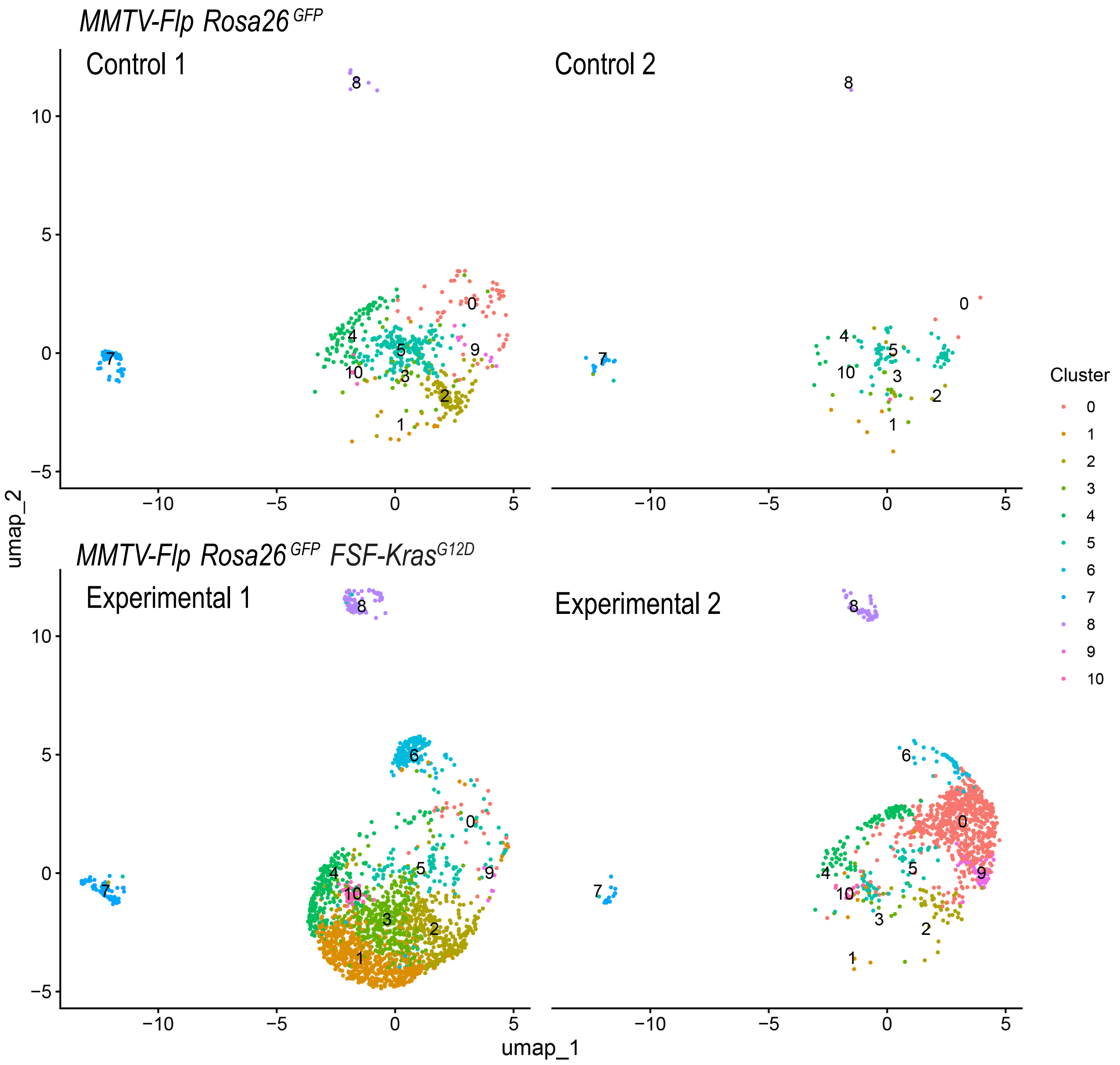

MMTV-Flp Rosa26 GFP
Control 1
Control 2
MMTV-Flp Rosa26 GFP FSF-KrasG12D
Experimental 1
Experimental 2
umap_2
umap_1
Cluster
0
1
2
3
4
5
6
7
8
9
10

Supplemental Figure S8. Emergence of a unique cellular population (cluster 6) exclusively identified in oncogenic KRAS-expressing samples.

Uniform Manifold Approximation and Projection (UMAP) plots of eleven transcriptionally distinct mammary epithelial cell states (clusters 0-10) in two independent datasets from *MMTV-Flp Rosa26-GFP* control females (upper panels) and two independent datasets from *MMTV-Flp Rosa26-GFP FSF-Kras*^{G12D} experimental females (lower panels).

Supplemental Fig. S9 Wicker et al.

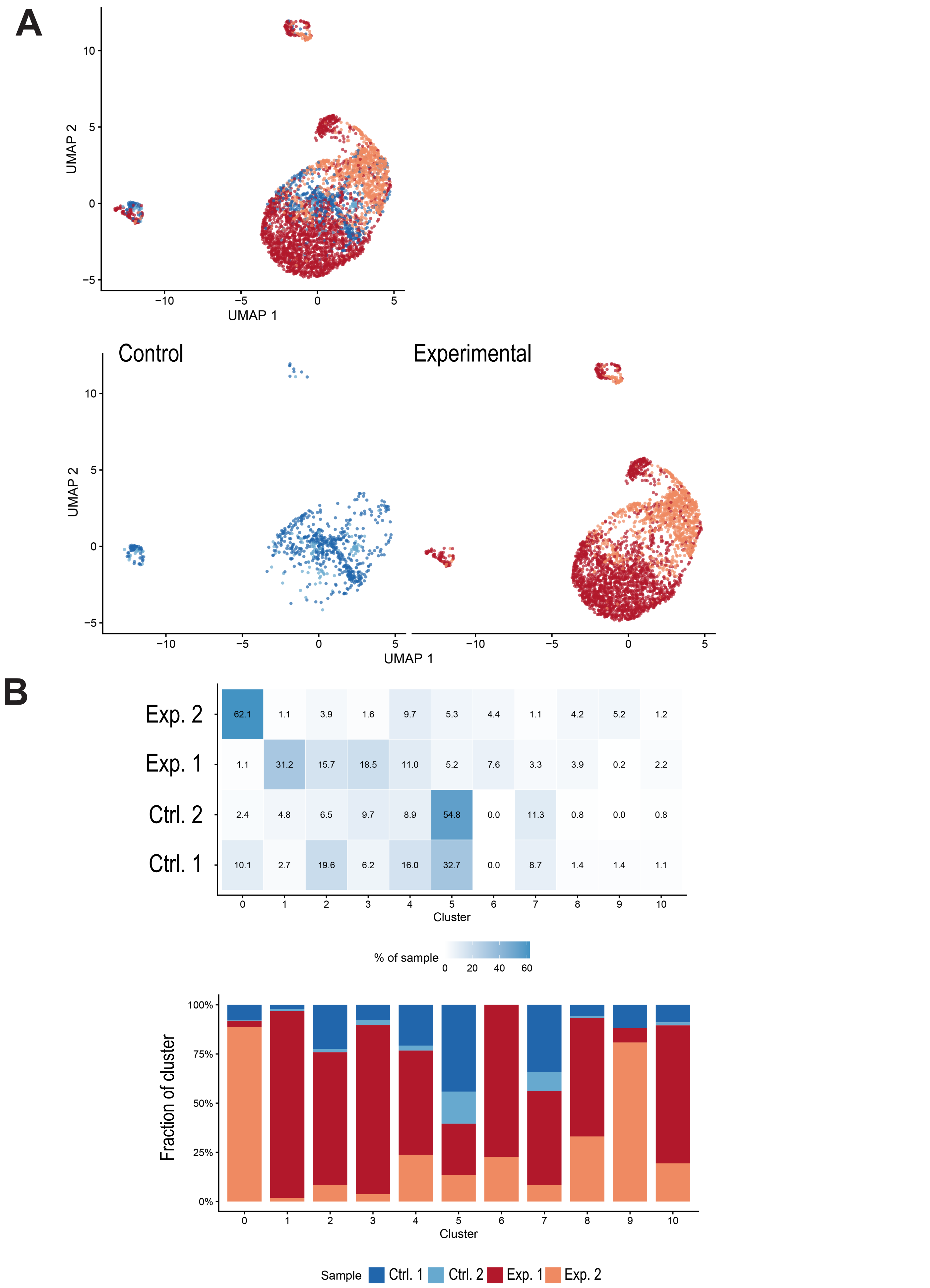

Supplemental Figure S9. The unique KRAS-associated cellular state (cluster 6) is consistently and exclusively identified across independent experimental libraries.

A. UMAP projections of mammary epithelial cells from two independently processed pooled control libraries (Ctrl. 1 and Ctrl. 2; blue shades) and two independently processed pooled KRAS-expressing experimental libraries (Exp. 1 and Exp. 2; red/orange shades). Cells are shown together (top) and separately by genotype (bottom).

B. Heatmap showing the percentage of epithelial cells assigned to each cluster within each library (top), and stacked bar plot showing the relative contribution of each library to each cluster (bottom). Cluster 6 was detected in both experimental libraries and was not present in either control library. These observations support recurrence across experimental libraries but do not establish reproducibility among individual mice or exclude all technical effects.

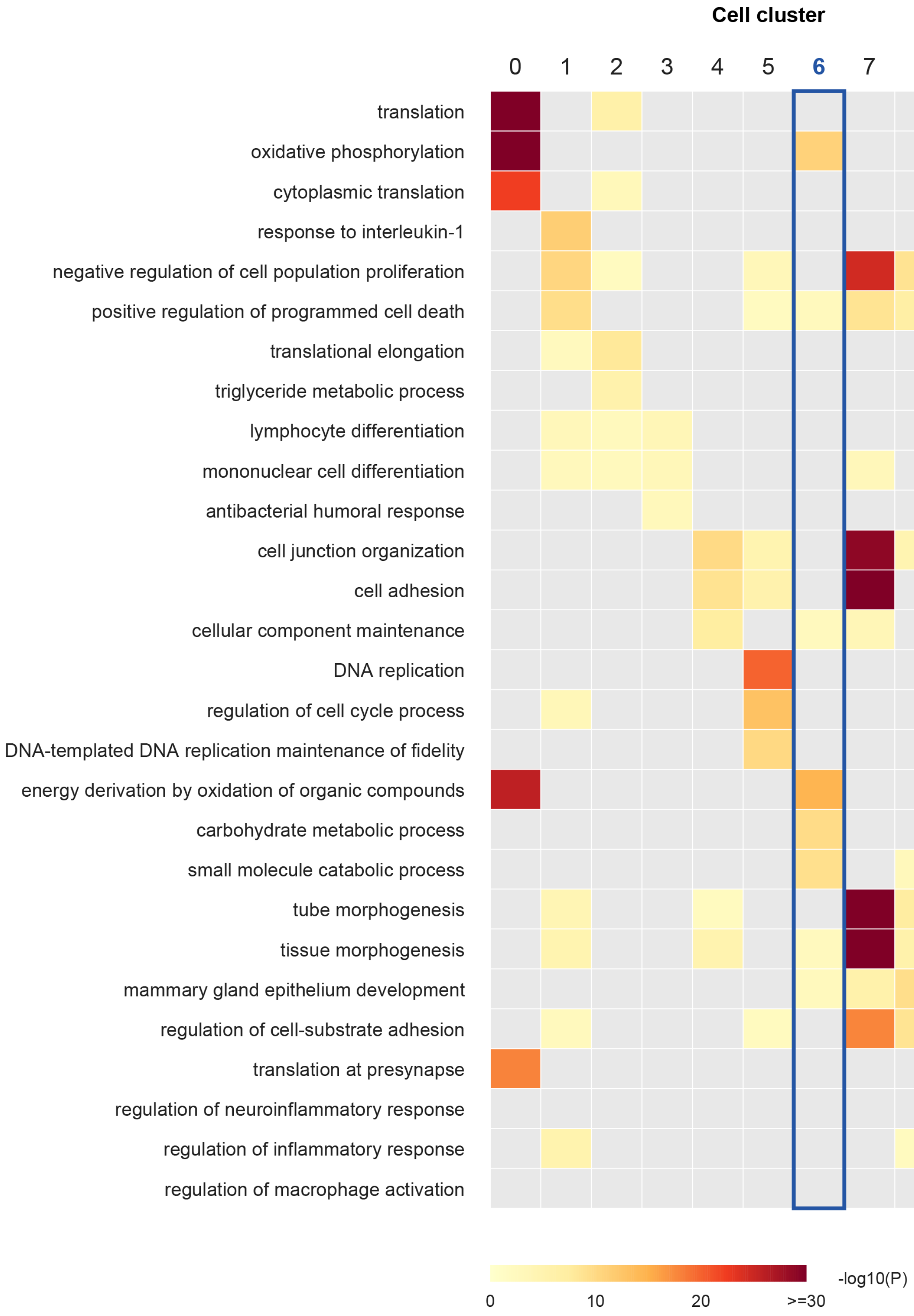
Cell cluster
0
1
2
3
4
5
6
7
8
9
10
translation
oxidative phosphorylation
cytoplasmic translation
response to interleukin-1
negative regulation of cell population proliferation
positive regulation of programmed cell death
translational elongation
triglyceride metabolic process
lymphocyte differentiation
mononuclear cell differentiation
antibacterial humoral response
cell junction organization
cell adhesion
cellular component maintenance
DNA replication
regulation of cell cycle process
DNA-templated DNA replication maintenance of fidelity
energy derivation by oxidation of organic compounds
carbohydrate metabolic process
small molecule catabolic process
tube morphogenesis
tissue morphogenesis
mammary gland epithelium development
regulation of cell-substrate adhesion
translation at presynapse
regulation of neuroinflammatory response
regulation of inflammatory response
regulation of macrophage activation
-log10(P)
0
10
20
>=30

Supplemental Figure S10. Gene Ontology (GO) enrichment analysis of biological processes across the 11 mammary epithelial cell states.

Gene Ontology (GO) analysis of molecular pathways enriched across all 11 cellular states in comparison to the KRAS-induced cluster 6 (boxed). Box colors indicate enrichment as -log10(*P-value*).

A

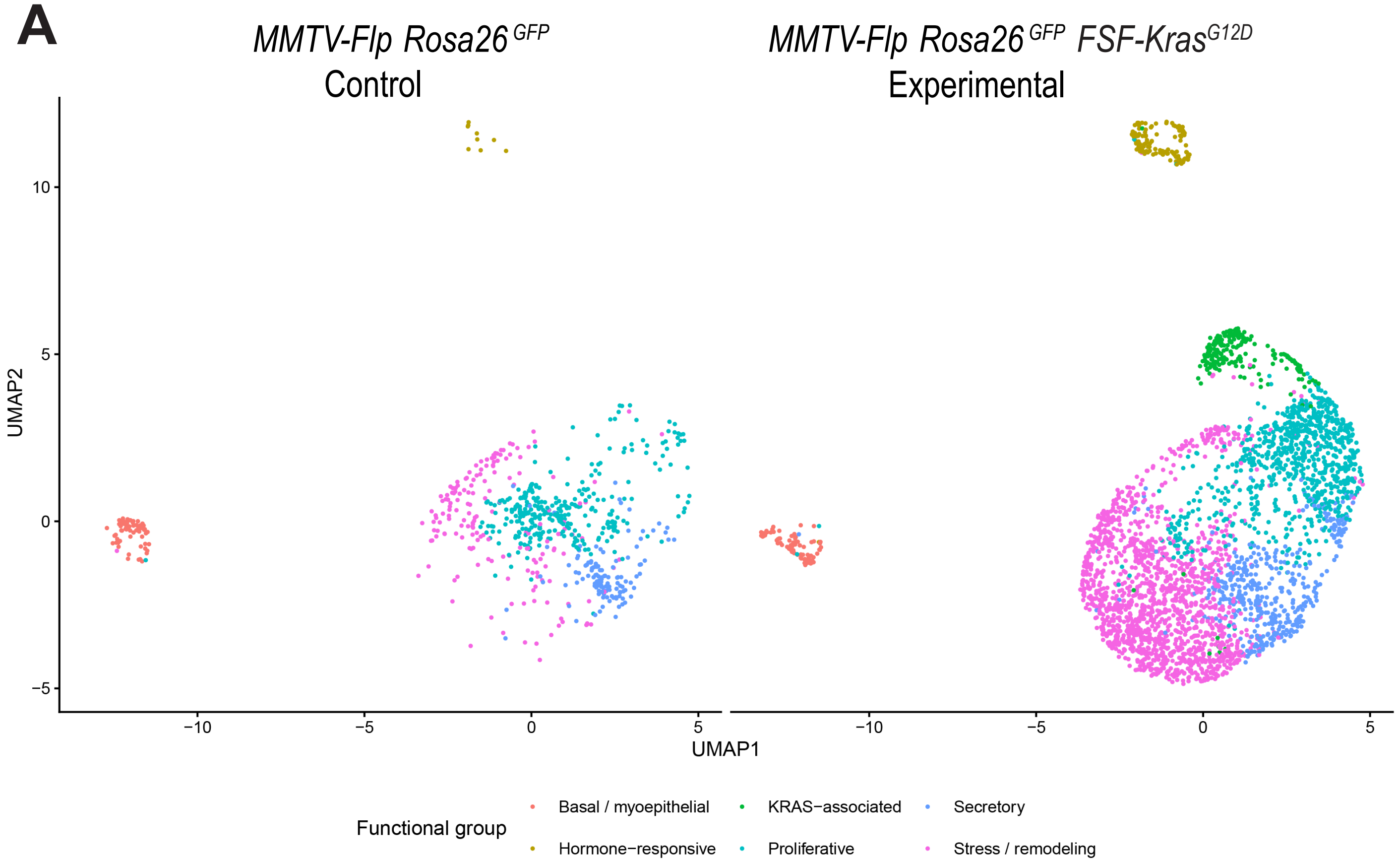
MMTV-Flp Rosa26 GFP
Control
MMTV-Flp Rosa26 GFP FSF-Kras G12D
Experimental
UMAP2
UMAP1
10
5
0
−5
−10
Functional group
Basal / myoepithelial
KRAS−associated
Secretory
Hormone−responsive
Proliferative
Stress / remodeling

B

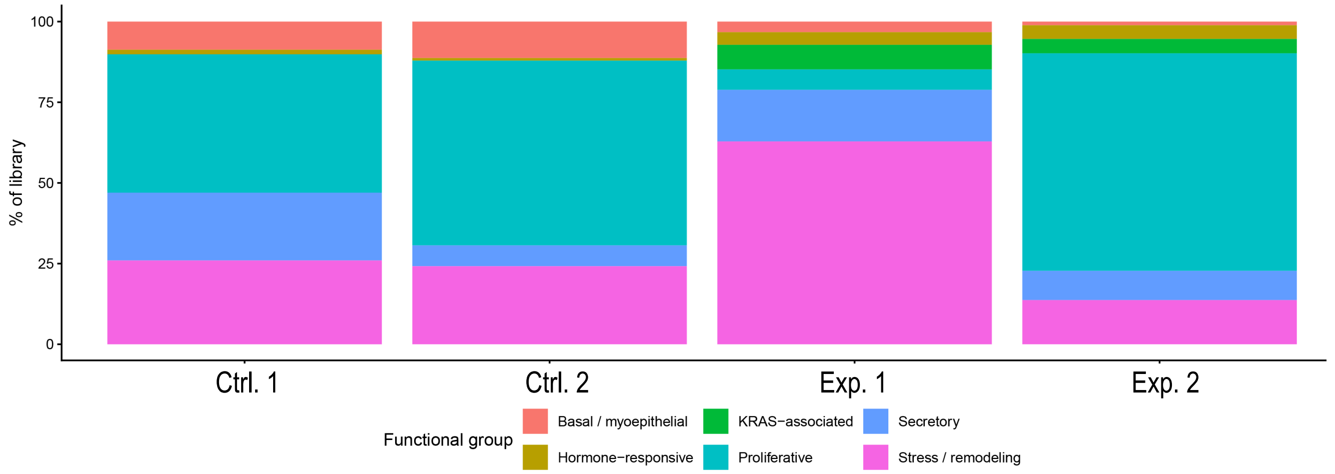
% of library
100
75
50
25
0
Ctrl. 1
Ctrl. 2
Exp. 1
Exp. 2
Functional group
Basal / myoepithelial
KRAS−associated
Secretory
Hormone−responsive
Proliferative
Stress / remodeling

Supplemental Figure S11. Unique presence of the oncogenic-permissive functional group (cluster 6, green) in experimental females expressing mutant KRAS.

A. UMAP plot of GFP-expressing epithelial cell clusters within functional groups as defined by the top 20 most differentially expressed genes in *MMTV-Flp Rosa26-GFP* control females (left) and *MMTV-Flp Rosa26-GFP FSF-Kras*^{G12D} experimental females (right).

B. Relative number of cells within each functional group across two independently processed libraries from *MMTV-Flp Rosa26-GFP* control females (Ctrl. 1/2) and two independently processed libraries from *MMTV-Flp Rosa26-GFP FSF-Kras*^{G12D} experimental females (Exp. 1/2).

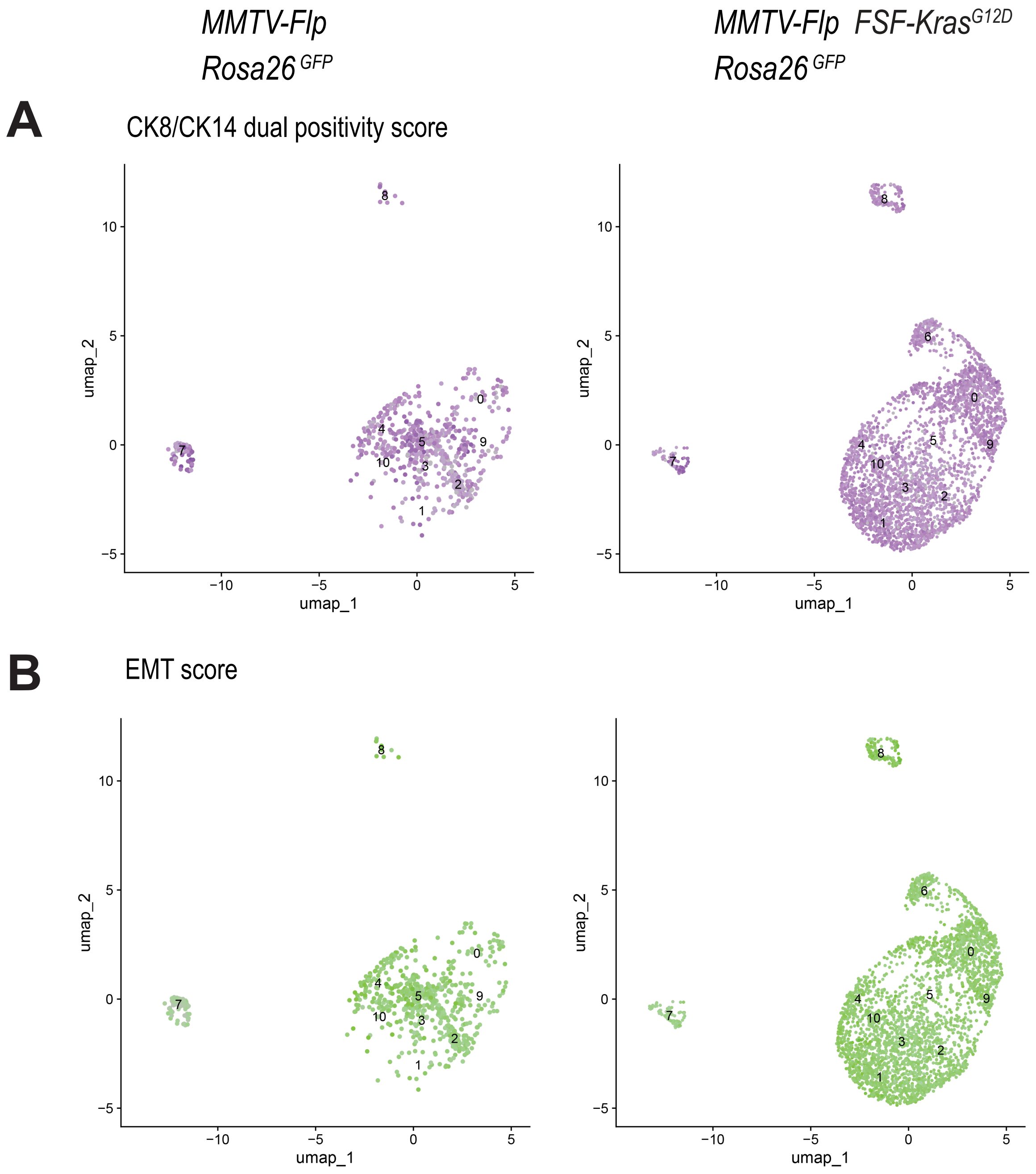
MMTV-Flp
Rosa26 GFP
MMTV-Flp FSF-Kras G12D
Rosa26 GFP
A
CK8/CK14 dual positivity score
B
EMT score
umap_1
umap_2

Supplemental Figure S12. The luminal–basal marker profile and high EMT signature scores suggest a pronounced degree of epithelial plasticity in mammary epithelial cells expressing oncogenic KRAS.

UMAP representation of the CK8/CK14 dual-positivity module score (**A**) and the epithelial-to-mesenchymal (EMT) module score (**B**) in GFP-positive mammary epithelial cells from *MMTV-Flp Rosa26-GFP* control females (left) and *MMTV-Flp Rosa26-GFP FSF-Kras*^{G12D} experimental females (right).

A

Breast-specific developmental signatures

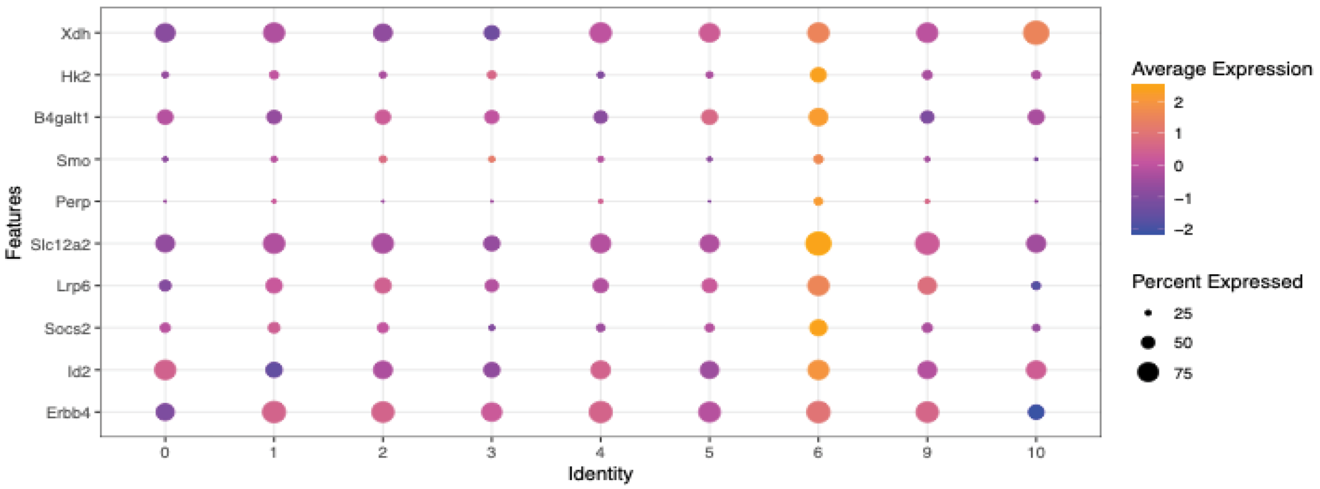

B

Lipid metabolism rewiring

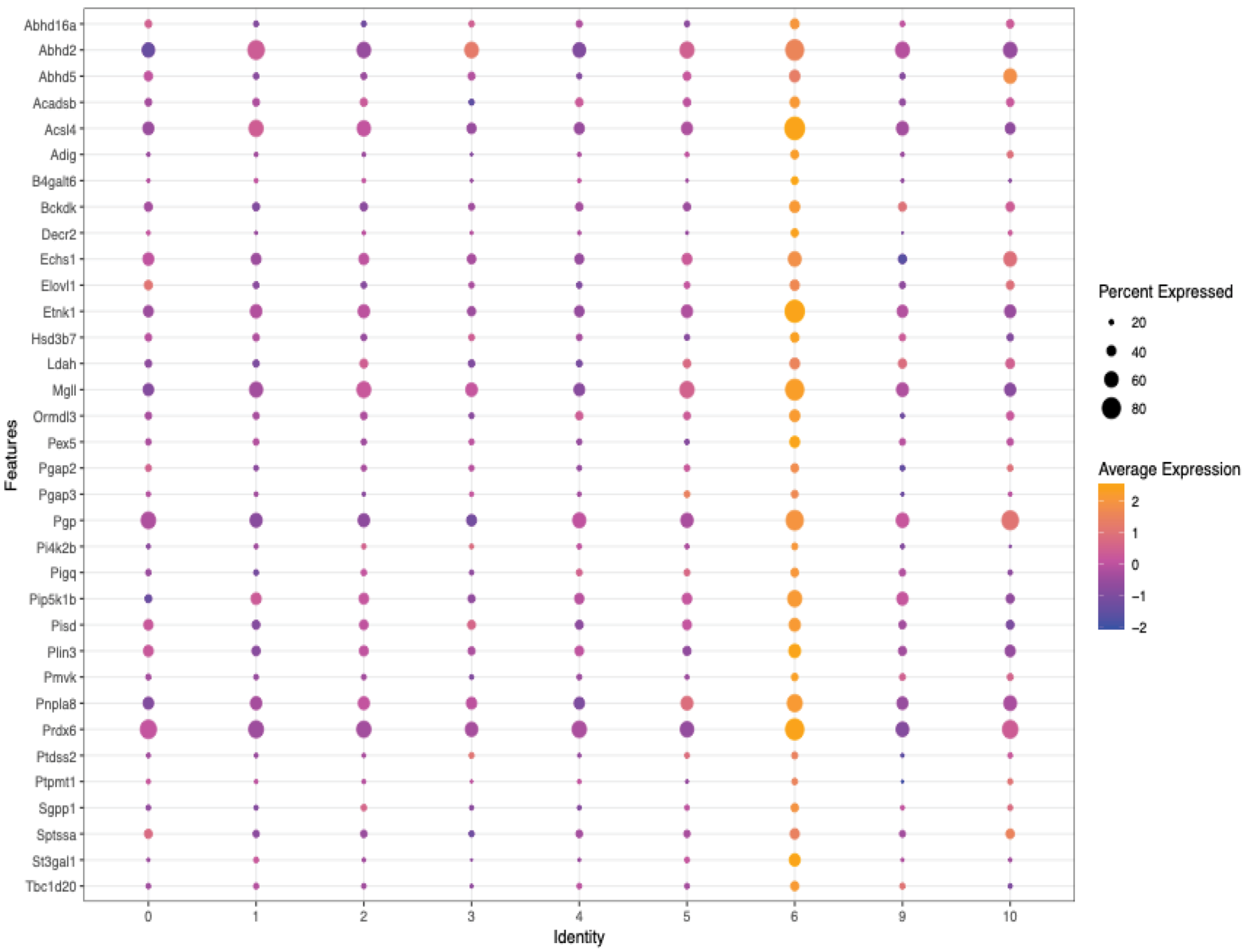

Supplemental Figure S13. The novel epithelial state (cluster 6) in response to oncogenic KRAS activation is characterized by enrichment of genes associated with mammary development and metabolic rewiring.

A. Dot plot illustrating the expression profiles of a 10-gene signature associated with mammary gland development across all 11 epithelial cell states identified in GFP-positive mammary epithelial cells isolated from *MMTV-Flp Rosa26-GFP FSF-Kras*^{G12D} females.

B. Dot plot depicting the expression profiles of genes associated with lipid metabolism across all 11 epithelial cell states. Dot color represents average gene expression; dot size represents the percentage of cells expressing each gene.

Supplemental Fig. S14

Wicker et al.

A

Breast cancer / oncogenic pathways

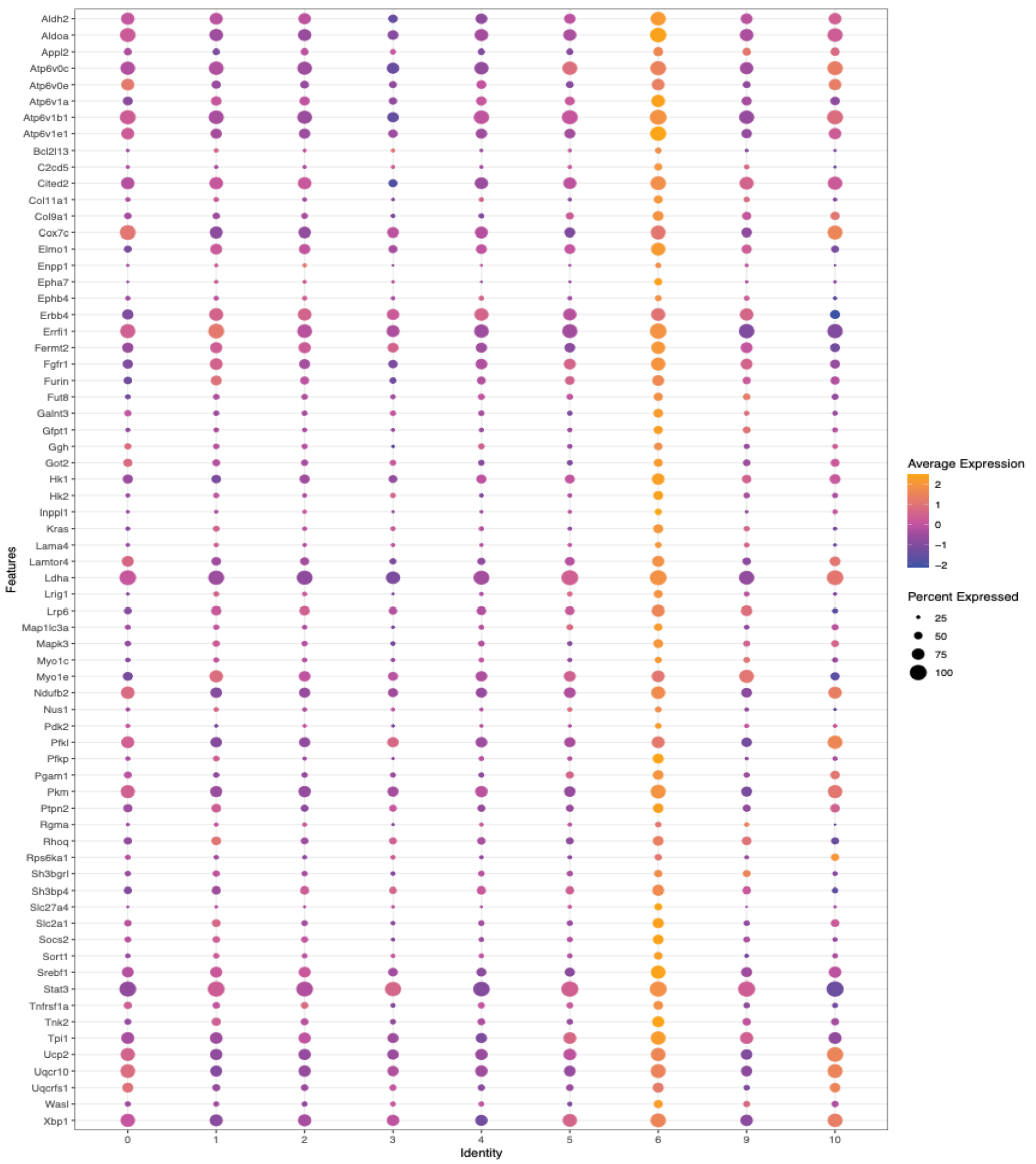

B

Metastasis and tumor progression

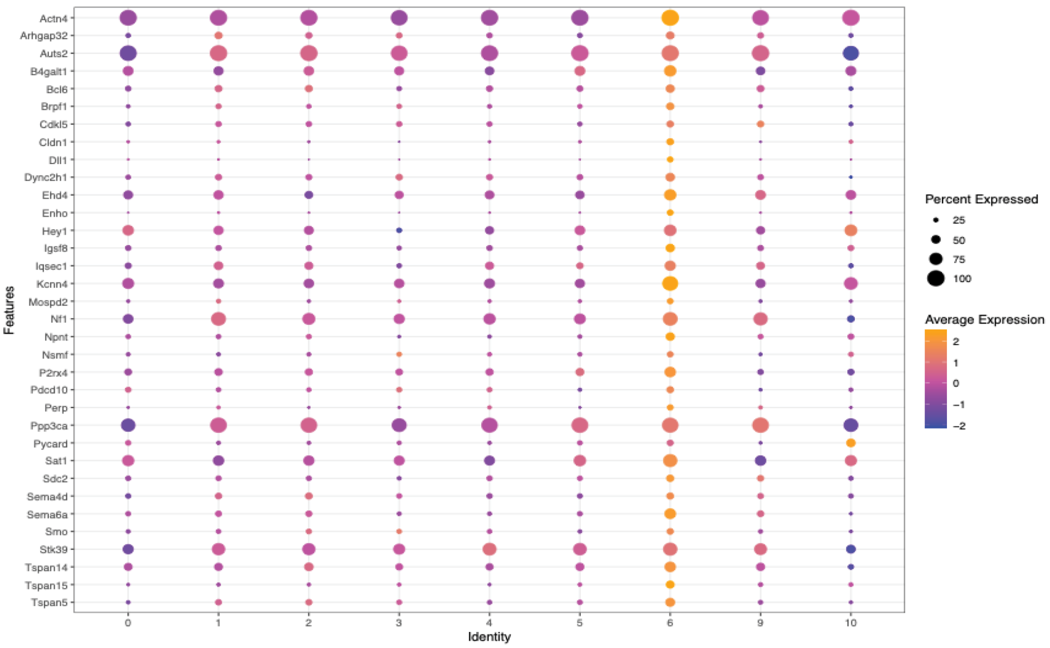

Supplemental Figure S14. Mammary epithelial cells induced by mutant KRAS in cluster 6 exhibit elevated expression of genes associated with oncogenic signaling and tumor progression.

A. Dot plot visualizing the expression profiles of genes associated with breast cancer and oncogenic signaling pathways across all 11 epithelial cell states identified among GFP-positive mammary epithelial cells isolated from *MMTV-Flp Rosa26-GFP FSF-Kras*^{G12D} females.

B. Dot plot displaying the expression profiles of genes associated with metastasis and tumor progression across all 11 epithelial cell states. Dot color represents average gene expression; dot size represents the percentage of cells expressing each gene.

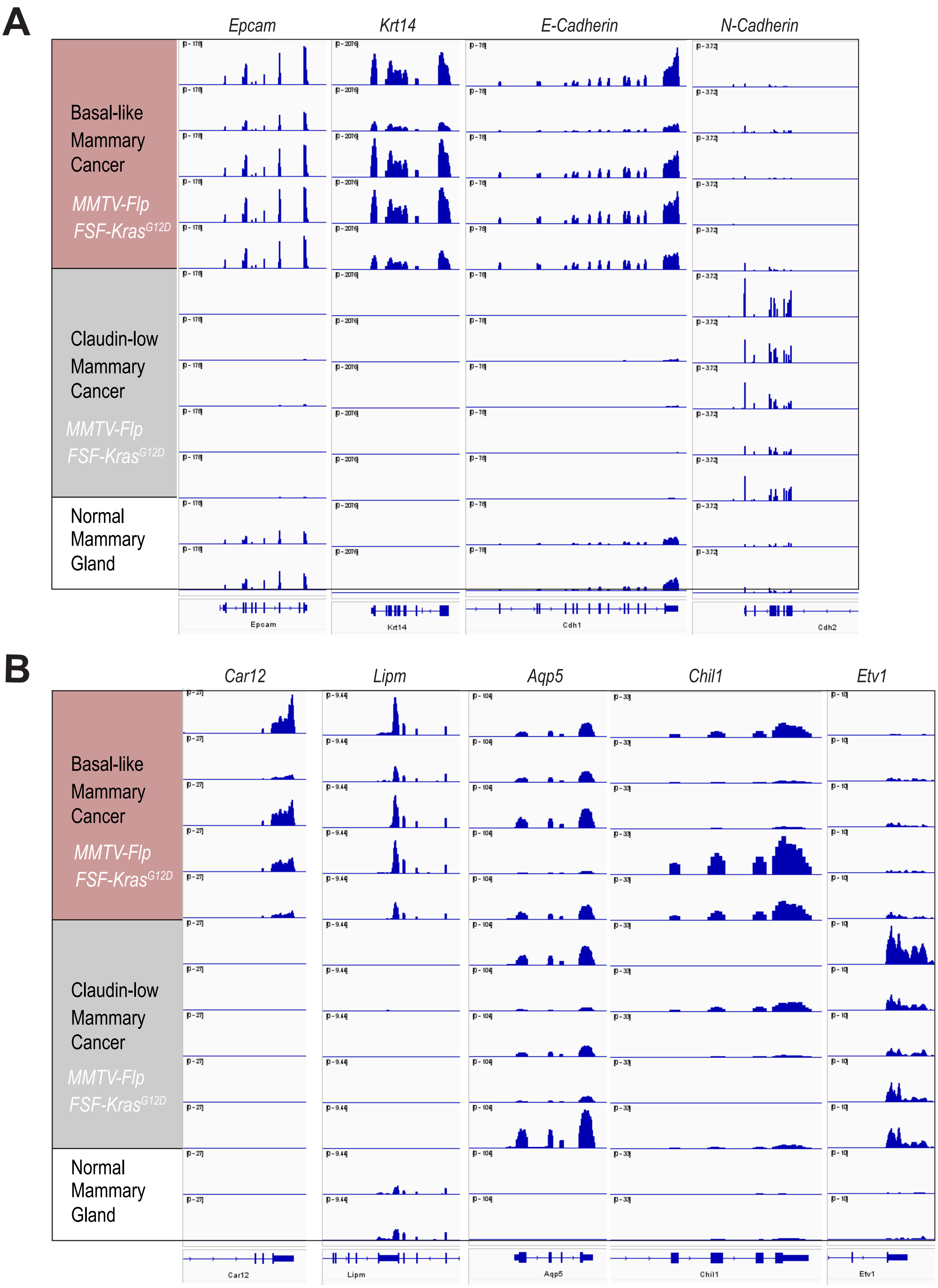
A
Epcam
Krt14
E-Cadherin
N-Cadherin
Basal-like Mammary Cancer
MMTV-Flp
FSF-Kras^{G12D}
Claudin-low Mammary Cancer
MMTV-Flp
FSF-Kras^{G12D}
Normal Mammary Gland
Epcam
Krt14
Cdh1
Cdh2
B
Car12
Lipm
Aqp5
Chil1
Etv1
Basal-like Mammary Cancer
MMTV-Flp
FSF-Kras^{G12D}
Claudin-low Mammary Cancer
MMTV-Flp
FSF-Kras^{G12D}
Normal Mammary Gland
Car12
Lipm
Aqp5
Chil1
Etv1

Supplemental Figure S15. Elevated expression of selected signature genes defining the novel transformation-competent mammary epithelial state (cluster 6) in mutant KRAS-induced basal-like and claudin-low mammary tumors.

A. RNA-seq coverage tracks showing normalized expression profiles of selected genes that discriminate five basal epithelial-like from five claudin-low (mesenchymal-like) mammary tumors and two normal mammary glands.

B. RNA-seq coverage tracks showing elevated expression of five cluster 6-defining genes in triple-negative mammary cancers that originated in *MMTV-Flp FSF-Kras*^{G12D} females. The plots illustrate the transcript coverage across individual exons.

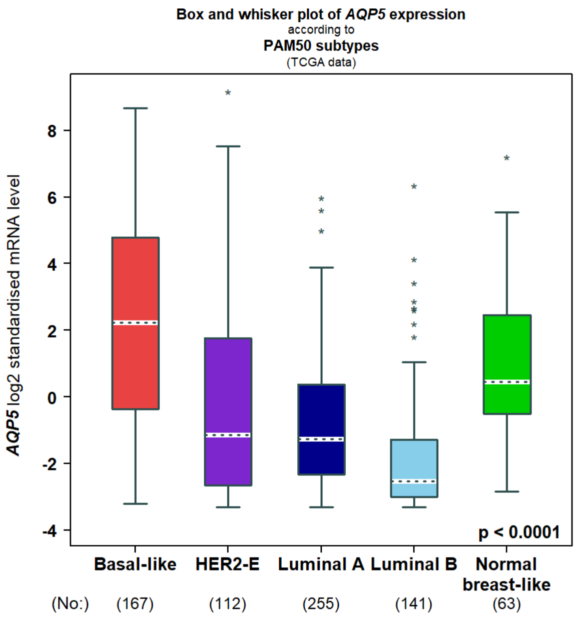

p(Welch) < 0.0001

Show Basal-like

Dunnett-Tukey-Kramer's test:

gene-expression comparisons			p-value
Basal-like	>	HER2-E	< 0.0001
Basal-like	>	Luminal A	< 0.0001
Basal-like	>	Luminal B	< 0.0001
Basal-like	>	Normal breast-like	< 0.01

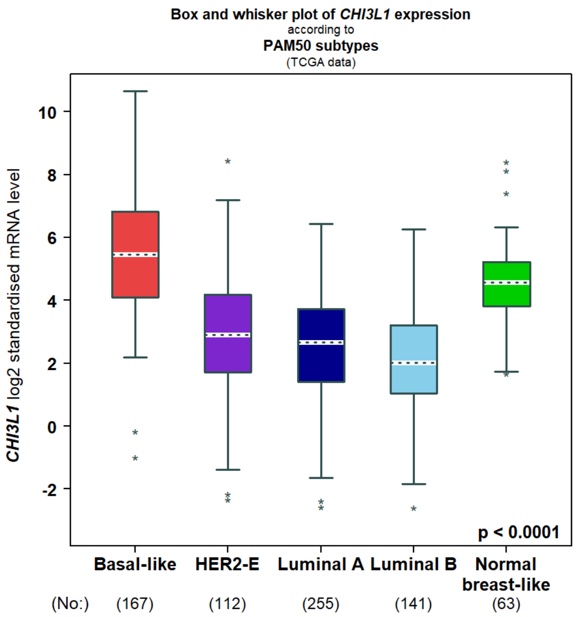

p(Welch) < 0.0001

Show Basal-like

Dunnett-Tukey-Kramer's test:

gene-expression comparisons			p-value
Basal-like	>	HER2-E	< 0.0001
Basal-like	>	Luminal A	< 0.0001
Basal-like	>	Luminal B	< 0.0001
Basal-like	>	Normal breast-like	< 0.0001

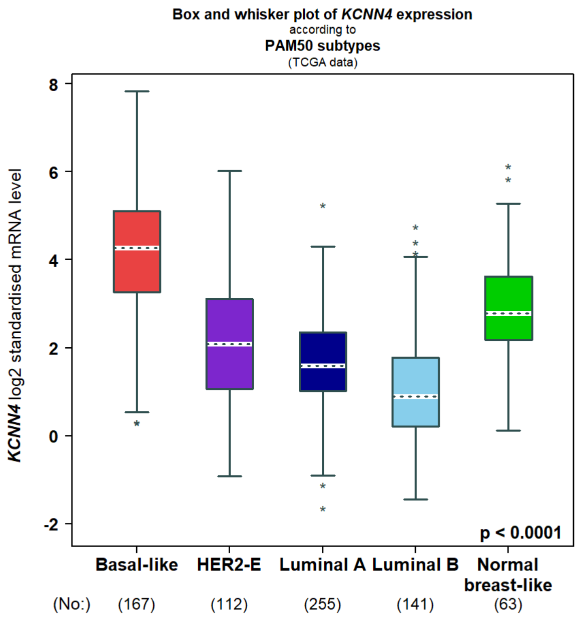

p(Welch) < 0.0001

Show Basal-like

Dunnett-Tukey-Kramer's test:

gene-expression comparisons			p-value
Basal-like	>	HER2-E	< 0.0001
Basal-like	>	Luminal A	< 0.0001
Basal-like	>	Luminal B	< 0.0001
Basal-like	>	Normal breast-like	< 0.0001

Supplemental Figure S16. Upregulated expression of selected genes defining the unique transformation-competent mammary epithelial state (cluster 6) across human breast cancer subtypes.

Box plots illustrate the significantly elevated expression of *AQP5, CHI3L1*, and *KCNN4* across five human molecular breast cancer subtypes (data source TCGA, Breast Cancer Gene-Expression Miner v5.3).

Supplemental Fig. S17

Wicker et al.

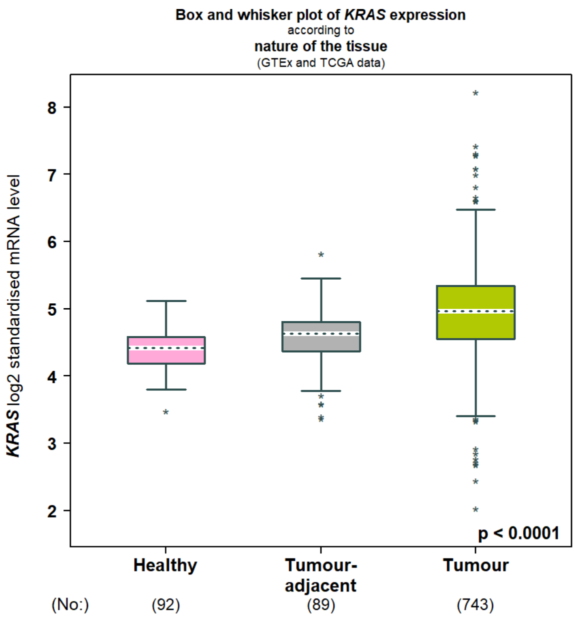

p(Welch) < 0.0001			
**Dunnett-Tukey-Kramer's test:**			
gene-expression comparisons			p-value
Tumour	>	Healthy	< 0.0001
Tumour	>	Tumour-adjacent	< 0.0001
Tumour-adjacent	>	Healthy	< 0.05

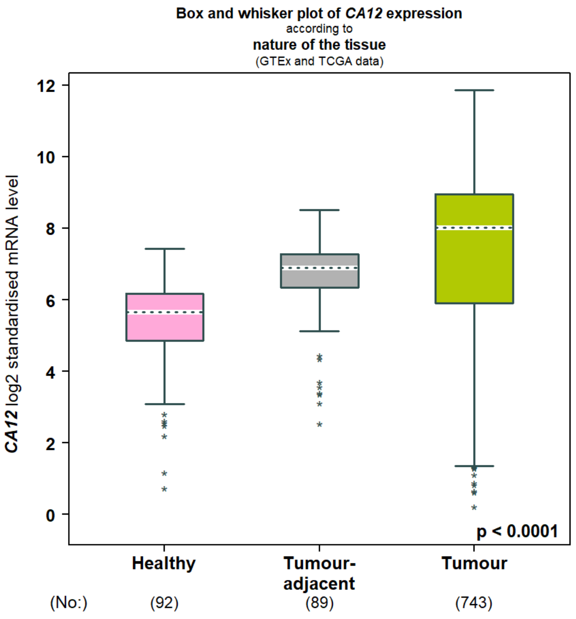

p(Welch) < 0.0001			
**Dunnett-Tukey-Kramer's test:**			
gene-expression comparisons			p-value
Tumour-adjacent	>	Healthy	< 0.0001
Tumour	>	Healthy	< 0.0001
Tumour	>	Tumour-adjacent	< 0.001

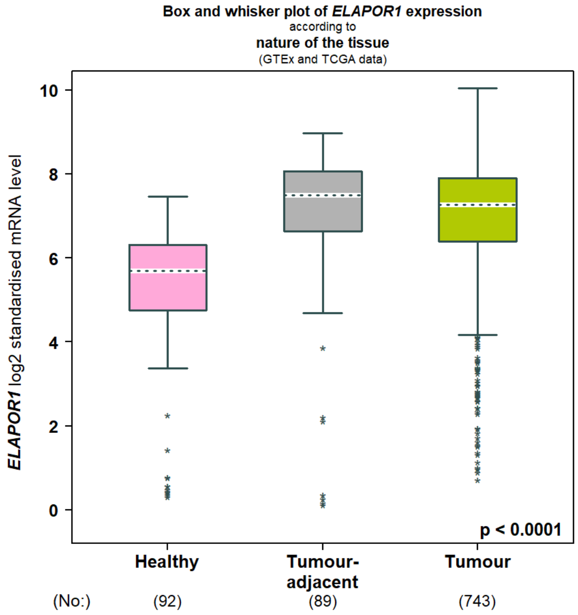

p(Welch) < 0.0001			
**Dunnett-Tukey-Kramer's test:**			
gene-expression comparisons			p-value
Tumour-adjacent	>	Healthy	< 0.0001
Tumour	>	Healthy	< 0.0001
Tumour	=	Tumour-adjacent	> 0.10

Supplemental Figure S17. Elevated expression of selected genes defining the KRAS-induced transformation-competent mammary epithelial state (cluster 6) in human breast tissue and tumors.

Box plots illustrating significantly elevated expression levels of *CA12* and *ELAPOR1* in tumor-adjacent normal breast tissue and breast cancers relative to normal breast tissue (data source: TCGA, Breast Cancer Gene-Expression Miner v5.3).

Supplemental Fig. S18 Wicker et al.

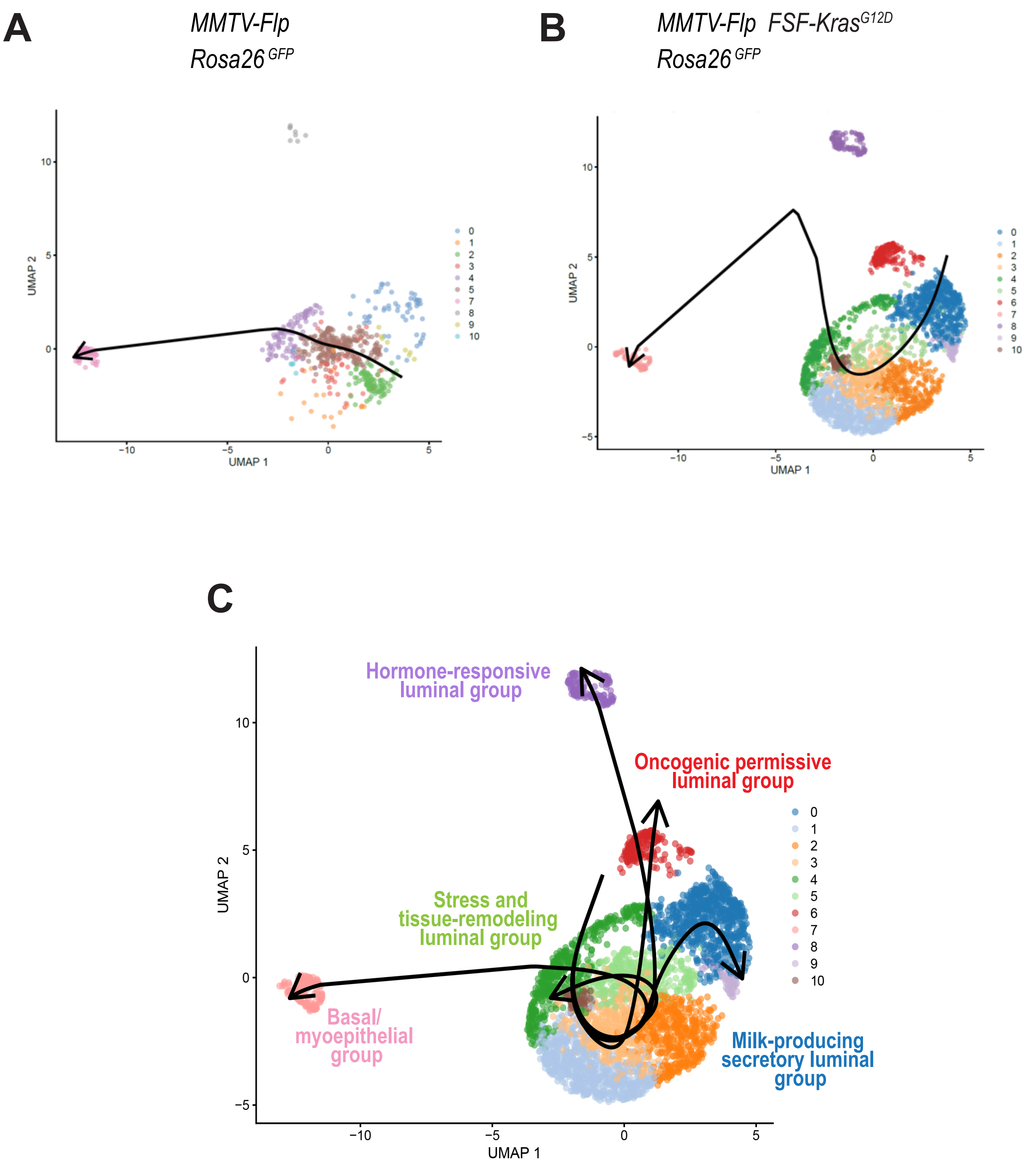

Supplemental Figure S18. Slingshot-inferred differentiation trajectories and pseudotime ordering of mammary epithelial cell states in control and KRAS-expressing mice.
Slingshot lineage and pseudotime analysis characterizing major mammary epithelial differentiation trajectories and assessing KRAS-associated differences across the 11 epithelial cell clusters identified in *MMTV-Flp Rosa26-GFP* control females (**A**) and *MMTV-Flp Rosa26-GFP FSF-Kras*^{G12D} experimental females (**B**). **C.** UMAP visualization of the combined control and experimental samples showing five inferred trajectories across the GFP-positive epithelial cell populations. Outlier cells were excluded prior to trajectory analysis. Black lines represent inferred differentiation lineages, and cells are colored by their established cluster identity.

Supplemental Fig. S19

Wicker et al.

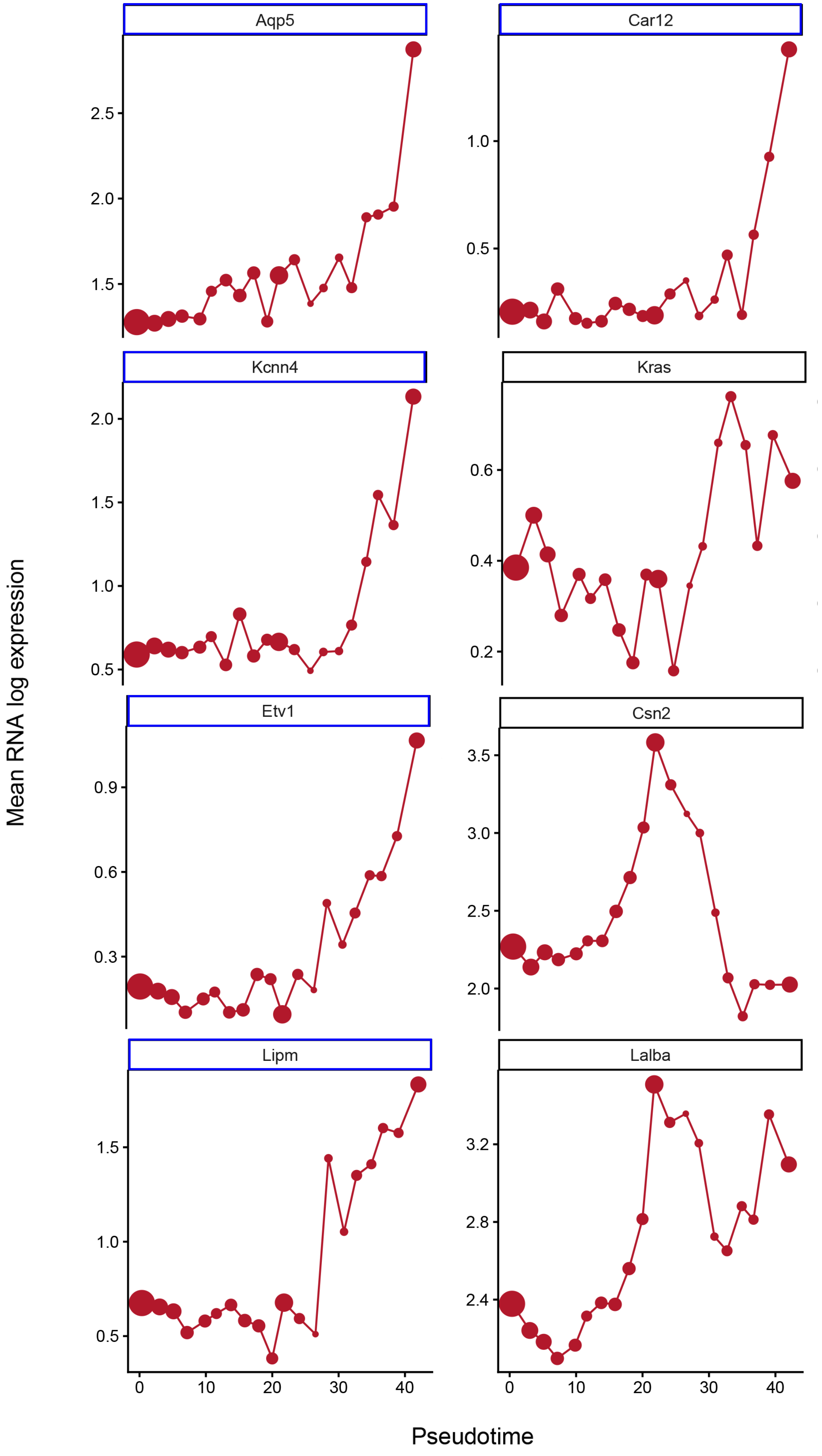

Supplemental Figure S19. Expression dynamics of genes defining the unique oncogenic-permissive mammary epithelial state (cluster 6) in KRAS-expressing mammary epithelial cells.
Gene expression wave plots for individual genes defining the unique KRAS-induced cellular state along the reconstructed experimental TSCAN path ending in cluster 6. Expression was summarized in 20 equal-width pseudotime bins, with point size indicating the number of cells per bin. Expression of the cluster 6-defining genes *Aqp5, Car12, Etv1*, *Kcnn4*, and *Lipm* (blue boxes) increases toward the cluster 6 end, whereas the secretory alveolar marker *Csn2* and *Lalba* are higher in the intermediate portion of the path. Following the trajectory of cluster 6-defining genes, *Kras* expression increases towards the cluster 6 endpoint.
Note: This is a descriptive reconstruction using the current software environment; pseudotime orientation does not establish lineage ancestry or temporal causality.

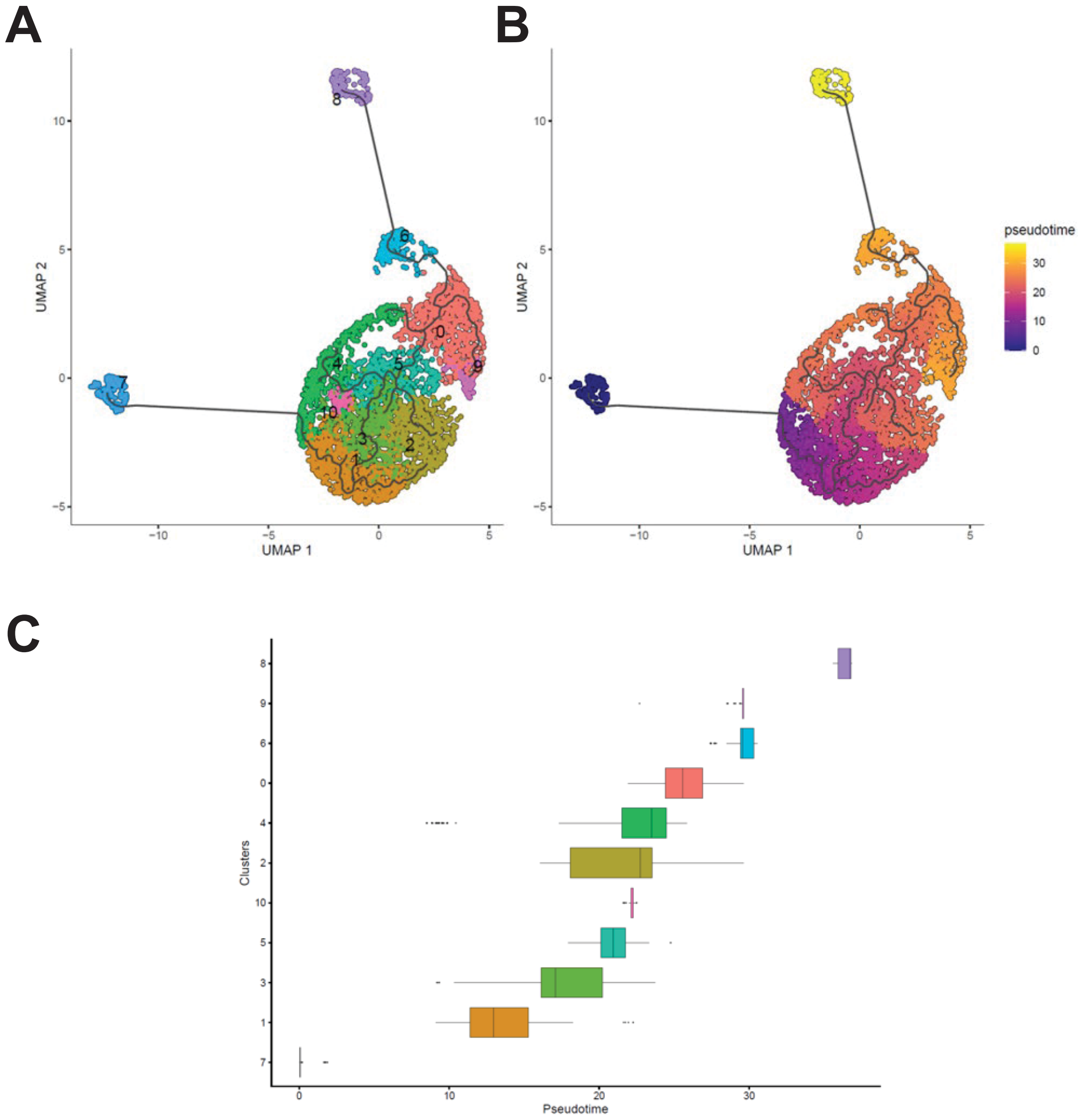
A
B
C
UMAP 2
UMAP 1
pseudotime
30
20
10
0
Clusters
Pseudotime

Supplemental Figure S20. Monocle3-inferred differentiation trajectories and pseudotime ordering of GFP-positive mammary epithelial cells from control and KRAS-expressing mice.

A. UMAP representation of the 11 mammary epithelial states according to cluster identity.
B. Monocle3-inferred trajectory colored according to pseudotime progression.
C. Distribution of epithelial cell states along inferred pseudotime, visualizing their relative positions within the reconstructed trajectory.

Supplementary Tables

Supplementary Table S1. PCR primers to genotype transgenes and genetically engineered alleles

Transgene/Allele	Primer #	Sequence (5' to 3')	bp
MMTV-Flp	2784	TTC TTC TGG TGC ATT CGA CAC TG	341 (wt)
vs wild-type Chr 11	2785	CCT CTG CCA TCT AGA ATG TGG	517 (tg)
	2786	ATC TTG AGG CTG CAA CTA AGC G	
FSF-Kras^{G12D}	2528	CAC CAG CTT CGG CTT CCT ATT	270 (wt)
	2529	AGC TAA TGG CTC TCA AAG GAA TGT A	350 (mut)
	2530	GCG AAG AGT TTG TCC TCA ACC	
Rosa26$^{CAG\text{-}FSF\text{-}GFP}$	2729	CCC AAA GTC GCT CTG AGT TGT TAT C	550 (wt)
	2730	GAA GGA GCG GGA GAA ATG GAT ATG	350 (mu)
	2731	CCA GGC GGG CCA TTT ACC GTA AG	

Supplementary Table S2. List of primary antibodies used for flow cytometric analysis

Antibody	Dilution	Source	Identifier
CD49f-PE/Cy7	1:40	BioLegend	Cat. #313621<br>RRID: AB_2561704
CD61-APC	1:100	Invitrogen	Cat. #17-0611-82<br>RRID: AB_2848288
EpCAM-PE	1:200	Invitrogen	Cat. 12-5791-82<br>RRID: AB_953615
7-AAD	1:100	BD Pharmingen	Cat. #559925<br>RRID: AB_2869266

Supplementary Table S3. List of primary antibodies used for lineage depletion

Antibody	Source	Identifier
Biotin-anti-CD31	Invitrogen	Cat. #13-0311-82<br>RRID: AB_466420
Biotin-anti-CD45	Invitrogen	Cat. #13-0451-82<br>RRID: AB_466446
Biotin-anti-CD34	Invitrogen	Cat. #13-0341-82<br>RRID: AB_466425
Biotin-anti-Ter119	Invitrogen	Cat. #MA5-17819<br>RRID: AB_2539203
Anti-biotin microbeads	Miltenyi Biotec	Cat. #130-090-485<br>RRID: AB_244365

Supplementary Table S4. PIP-seq library index and adapter sequence summary for sequencing

**Sample ID**	**Library i7 index**	**i7sequence** (from sample sheet)	**i7 adapter sequence**	**Library i5 index**	**i5 adapter sequence**
Control #1	706	TAGGCATG	CATGCCTA	506	ACTGCATA
Control #2	705	GGACTCCT	AGGAGTCC	505	GTAAGGAG
Experiment #1	701	TAAGGCGA	TCGCCTTA	501	TAGATCGC
Experiment #2	708	CAGAGAGG	CCTCTCTG	508	CTAAGCCT

Addendum

Flow cytometric images, gating strategy

A

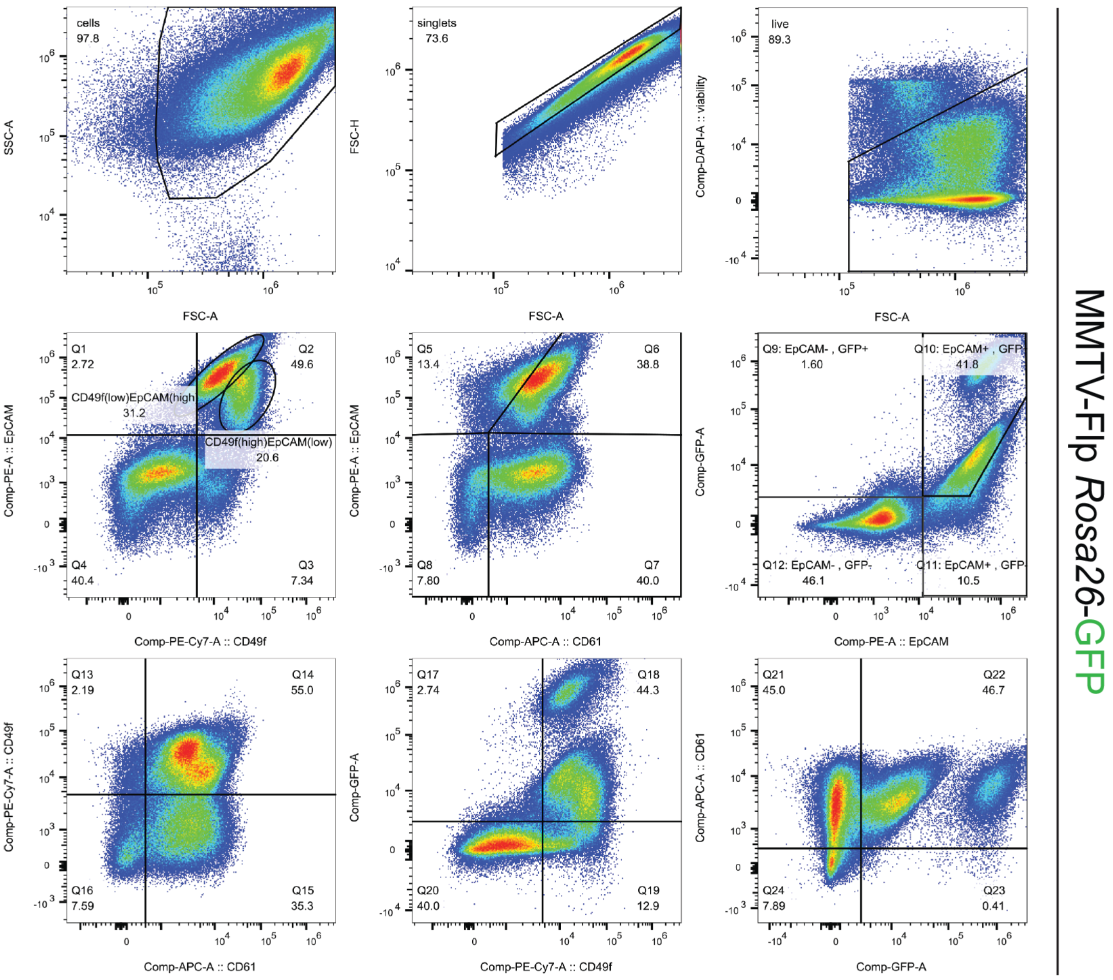
cells
97.8
SSC-A
FSC-A
singlets
73.6
FSC-H
FSC-A
live
89.3
Comp-DAPI-A :: viability
FSC-A
Q1
2.72
Q2
49.6
CD49f(low)EpCAM(high)
31.2
CD49f(high)EpCAM(low)
20.6
Q4
40.4
Q3
7.34
Comp-PE-A :: EpCAM
Comp-PE-Cy7-A :: CD49f
Q5
13.4
Q6
38.8
Q8
7.80
Q7
40.0
Comp-PE-A :: EpCAM
Comp-APC-A :: CD61
Q9: EpCAM- , GFP+
1.60
Q10: EpCAM+ , GFP+
41.8
Q12: EpCAM- , GFP-
46.1
Q11: EpCAM+ , GFP-
10.5
Comp-GFP-A
Comp-PE-A :: EpCAM
Q13
2.19
Q14
55.0
Q16
7.59
Q15
35.3
Comp-PE-Cy7-A :: CD49f
Comp-APC-A :: CD61
Q17
2.74
Q18
44.3
Q20
40.0
Q19
12.9
Comp-GFP-A
Comp-PE-Cy7-A :: CD49f
Q21
45.0
Q22
46.7
Q24
7.89
Q23
0.41
Comp-APC-A :: CD61
Comp-GFP-A
MMTV-Flp Rosa26-GFP

B

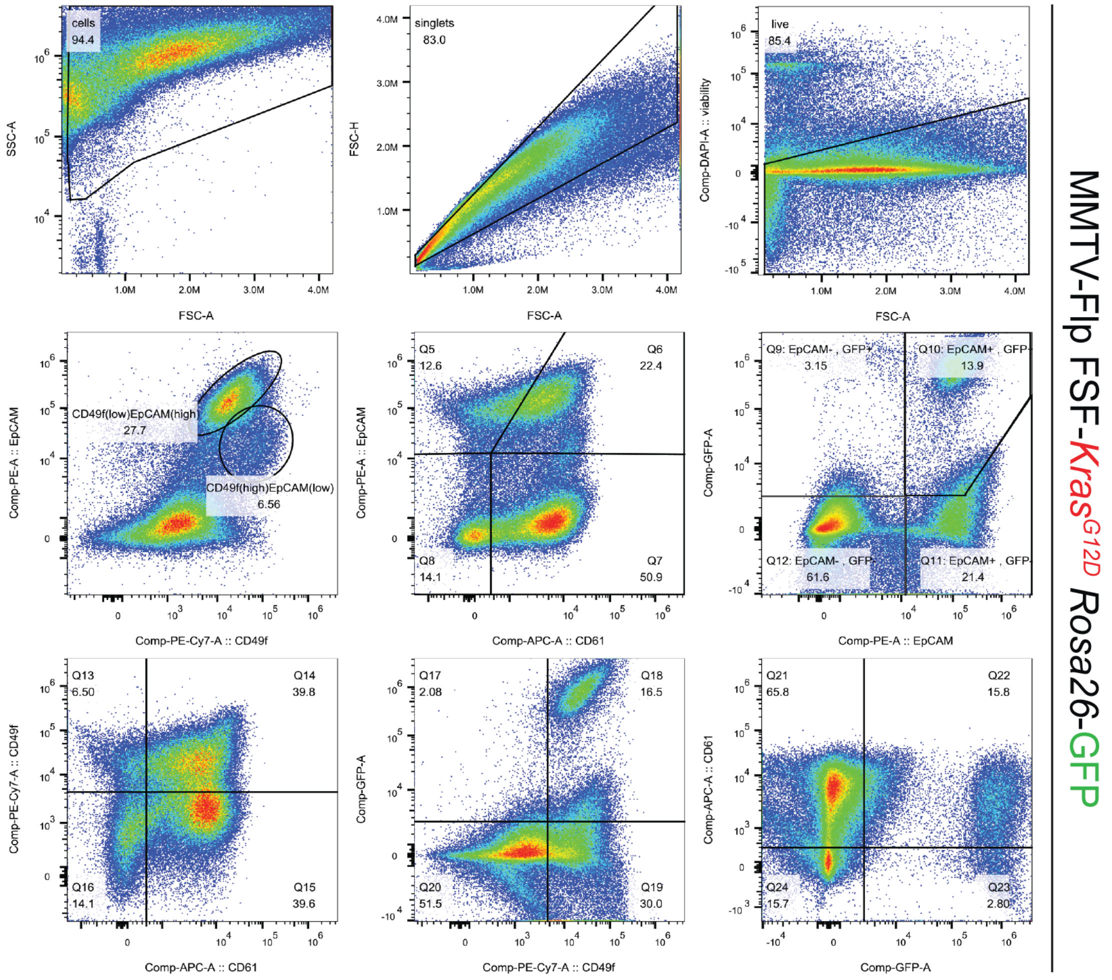
cells
94.4
SSC-A
FSC-A
singlets
83.0
FSC-H
FSC-A
live
85.4
Comp-DAPI-A :: viability
FSC-A
CD49f(low)EpCAM(high)
27.7
CD49f(high)EpCAM(low)
6.56
Comp-PE-A :: EpCAM
Comp-PE-Cy7-A :: CD49f
Q5
12.6
Q6
22.4
Q8
14.1
Q7
50.9
Comp-PE-A :: EpCAM
Comp-APC-A :: CD61
Q9: EpCAM- , GFP+
3.15
Q10: EpCAM+ , GFP+
13.9
Q12: EpCAM- , GFP-
61.6
Q11: EpCAM+ , GFP-
21.4
Comp-GFP-A
Comp-PE-A :: EpCAM
Q13
6.50
Q14
39.8
Q16
14.1
Q15
39.6
Comp-PE-Cy7-A :: CD49f
Comp-APC-A :: CD61
Q17
2.08
Q18
16.5
Q20
51.5
Q19
30.0
Comp-GFP-A
Comp-PE-Cy7-A :: CD49f
Q21
65.8
Q22
15.8
Q24
15.7
Q23
2.80
Comp-APC-A :: CD61
Comp-GFP-A
MMTV-Flp FSF-Kras^{G12D} Rosa26-GFP
